# Hexapod Assassins’ Potion: Venom Composition and Bioactivity from the Eurasian Assassin Bug *Rhynocoris iracundus*

**DOI:** 10.3390/biomedicines9070819

**Published:** 2021-07-14

**Authors:** Nicolai Rügen, Timothy P. Jenkins, Natalie Wielsch, Heiko Vogel, Benjamin-Florian Hempel, Roderich D. Süssmuth, Stuart Ainsworth, Alejandro Cabezas-Cruz, Andreas Vilcinskas, Miray Tonk

**Affiliations:** 1 Department of Bioresources, Fraunhofer Institute for Molecular Biology and Applied Ecology, Ohlebergsweg 12, 35392 Giessen, Germany; nicolai.ruegen@gmail.com (N.R.); Andreas.Vilcinskas@agrar.uni-giessen.de (A.V.); 2 Department of Biotechnology and Biomedicine, Technical University of Denmark, 2800 Kongens Lyngby, Denmark; tpaje@dtu.dk; 3 Research Group Mass Spectrometry/Proteomics, Max Planck Institute for Chemical Ecology, Hans-Knoell-Strasse 8, 07745 Jena, Germany; nwielsch@ice.mpg.de; 4 Department of Entomology, Max Planck Institute for Chemical Ecology, Hans-Knöll-Straße 8, 07745 Jena, Germany; hvogel@ice.mpg.de; 5 Department of Chemistry, Technische Universität Berlin, Strasse des 17. Juni 124, 10623 Berlin, Germany; benjamin.hempel@charite.de (B.-F.H.); suessmuth@chem.tu-berlin.de (R.D.S.); 6 BIH Center for Regenerative Therapies BCRT, Charité—Universitätsmedizin Berlin, 13353 Berlin, Germany; 7 Centre for Snakebite Research and Interventions, Department of Tropical Disease Biology, Liverpool School of Tropical Medicine, Liverpool L3 5QA, UK; stuart.ainsworth@lstmed.ac.uk; 8 UMR BIPAR, Laboratoire de Santé Animale, Anses, INRAE, Ecole Nationale Vétérinaire d’Alfort, F-94700 Maisons-Alfort, France; alejandro.cabezas@vet-alfort.fr; 9 Institute for Insect Biotechnology, Justus Liebig University of Giessen, Heinrich-Buff-Ring 26-32, 35392 Giessen, Germany; 10 LOEWE Centre for Translational Biodiversity Genomics (LOEWE-TBG), Senckenberganlage 25, 60325 Frankfurt, Germany

**Keywords:** assassin bugs, venom, transcriptomics, proteomics, bioactivity, paralysis, cytolysis, antibacterial, neurolysis

## Abstract

Assassin bug venoms are potent and exert diverse biological functions, making them potential biomedical goldmines. Besides feeding functions on arthropods, assassin bugs also use their venom for defense purposes causing localized and systemic reactions in vertebrates. However, assassin bug venoms remain poorly characterized. We collected the venom from the assassin bug *Rhynocoris iracundus* and investigated its composition and bioactivity in vitro and in vivo. It caused lysis of murine neuroblastoma, hepatoma cells, and healthy murine myoblasts. We demonstrated, for the first time, that assassin bug venom induces neurolysis and suggest that it counteracts paralysis locally via the destruction of neural networks, contributing to tissue digestion. Furthermore, the venom caused paralysis and melanization of *Galleria mellonella* larvae and pupae, whilst also possessing specific antibacterial activity against *Escherichia coli*, but not *Listeria grayi* and *Pseudomonas aeruginosa*. A combinatorial proteo-transcriptomic approach was performed to identify potential toxins responsible for the observed effects. We identified neurotoxic Ptu1, an inhibitory cystin knot (ICK) toxin homologous to ω-conotoxins from cone snails, cytolytic redulysins homologous to trialysins from hematophagous kissing bugs, and pore-forming hemolysins. Additionally, chitinases and kininogens were found and may be responsible for insecticidal and cytolytic activities. We demonstrate the multifunctionality and complexity of assassin bug venom, which renders its molecular components interesting for potential biomedical applications.

## 1. Introduction

Venoms typically consist of a plethora of highly diverse toxins that affect a complex range of physiological targets [1]. Consequently, venom components have become highly specialized with the ability to perform complex and intricate biochemical tasks within their target organism [2]. This ability to precisely manipulate specific organismal functions presents a biochemical gold mine of bio-active compounds that can be developed towards therapeutic or biotechnological applications [3,4].

The repurposing of venom toxins has been highly successful in the development of novel analgesics [5], diabetes drugs [6], and blood pressure modulators [7]. Due to their potential applicability, venom toxins are receiving significant attention to discover candidates as therapeutics for many other diseases [8,9]. Particularly, the rich profile of active venom molecules, often found in diverse venoms, present promising molecules for oncological studies [10,11].

Toxins from venomous animals have also been investigated as therapeutic antibacterial candidates in the face of increasing antibiotic resistance and as alternatives for chemical insecticides [12,13]. However, to date, the focus of such investigations has primarily been on toxins from ‘classical’ venomous animals, such as snakes, scorpions, and spiders, with only few studies investigating diverse insect venoms [14,15].

The suborder heteroptera with more than 40,000 described species deserve further attention, since among these numerous species at least some possess potent venoms [16,17]. Notably, heteropterans’ saliva has evolved in response to different trophic specializations [18]. Kissing bugs (Heteroptera: *Triatominae*) are hematophagous species specialized on blood meals and their saliva possess local anesthetic effects enabling stealth feeding [19,20]. While phytophagous hemipteran have specialized to feed on various plant tissues (e.g., vascular fluids, cell contents, and seeds) [21,22], assassin bugs (Heteroptera: *Reduviidae*) are zoophagous and use their venom to paralyze and liquefy their invertebrate prey [23]. Their venom can also have severe effects on vertebrates, including pain, muscle paralysis, hemorrhage, and even death of mice due to respiratory paralysis [24,25]. Reduviids are generalistic predators and their venoms are not species-specific, making them an interesting source of bio-active compounds which could be used in a medical or agronomical context (e.g., as insecticides).

It was previously reported from the harpactorine assassin bug *Pristhesancus plagipennis* and the red-spotted assassin bug *Platymeris rhadamanthus* that their venom comprises numerous enzymes, putative pore-forming toxins, and peptides [26,27]. Moreover, venom gland reconstructions recently revealed three distinct venom gland compartments: posterior main gland (PMG), anterior main gland (AMG), and accessory gland (AG), each containing venom with distinct functions [25]. *P. plagipennis* PMG venom, which can be elicited by electrostimulation, paralyzed and killed their prey, whilst AMG venom, extracted via harassment, did not paralyze prey in the studied species [25]. However, paralytic effects of AMG venom were found in other assassin bug species [28]. AMG venom was suggested to serve defensive purposes and might deter common predators such as birds and small mammals [18,25]. Furthermore, despite the small size of assassin bugs, their venom is potent and medically significant to humans, causing symptoms such as sharp pain, edema, and fever [29]. Although assassin bugs specialized to feed on invertebrates, the cases reporting negative effects on humans demonstrate the bioactivity and complexity of their venom, making it an untapped and valuable source for biomedical applications.

**Figure 1:**
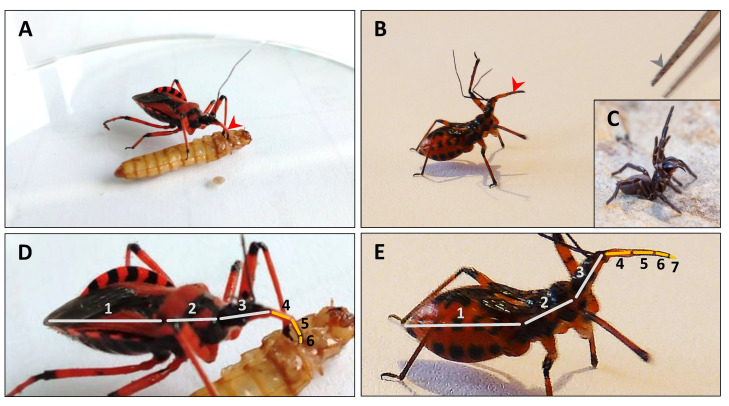
*Rhynocoris iracundus* using its proboscis for feeding or defense purposes. (**A**) *R. iracundus* using its proboscis (red arrowhead) to feed on a *Tenebrio molitor* larva. The front legs are used to grab and hold its prey. (**B**) *R. iracundus* in defense position with its elevated front legs and long proboscis (red arrowhead), ready to attack entomological forceps (gray arrowhead). (**C**) Defense position of a Blue Mountains funnel-web spider *Hadronyche versuta* with elevated front legs and chelicerae (Photo credits: with permission of Greg Bourke). (**D**,**E**) Alignment of main body and proboscis segments during feeding and defense, respectively. Body segments are shown with gray lines: abdomen (1), thorax (2), head (3), and proboscis segments are displayed as yellow lines (4–7).

Among reduviids, the assassin bug *Rhynocoris iracundus* has a particularly conspicuous behavior. Instead of hiding, *R. iracundus* is highly exposed to various predators since it ranges freely and prefers to ambush prey on flowers (personal observation). *R. iracundus* can use its proboscis to inject venom and for feeding purposes (Figure 1A) or defense (Figure 1B). During defense, *R. iracundus* displays similar behaviors and body position as typically observed in arachnids (Figure 1C). During feeding the main body segments are aligned in a position allowing an easy flow of liquefied prey content from the proboscis to the digestive organs (Figure 1D). During defense or prey attack, the thorax and abdomen are lifted allowing the assassin bug to raise and strike with its proboscis. The segments of the latter are then aligned which allows the assassin bug to transfer the full striking energy emerging from the main body to the tip of the proboscis (Figure 1E). As a first line of defense, some assassin bug species have developed additional protective morphological features, such as dorsal crests as in *Arilus cristatus*, a thorn bush as in *Sinea diadema*, or a crown of prominent thorns as in *Platymeris biguttatus* and *Psytalla horrida*. The assassin bug *R. iracundus* shares aposematic coloration, but lacks these morphological features, and therefore relies on its venom for protection.

Here, we study *R. iracundus* venom in detail and assess its molecular composition and functional characteristics for the first time. We tested the range of activity of *R. iracundus* venom on mammalian, insect, and bacterial cells, and we conducted in vivo studies in insects. To identify the compounds causing the observed effects, we performed a combinatorial proteo-transcriptomic analysis of the venom glands and the harvested venom. This study highlights the broad activity spectrum and potency of *R. iracundus* venom and identifies key candidates for potential therapeutic or insecticidal use.

## 2. Materials and Methods

### 2.1. Assassin Bug Collection, Rearing, and Venom Collection

*R. iracundus* nymphs were collected from North Rhine-Westphalia, Germany, with permission granted from the nature conservation authority as part of the County Government of Rhineland-Palatinate (Obere Naturschutzbehörde, permission No. 425-104.1713). *R. iracundus* used in this study were kept on a diet composed of meal worm *Tenebrio molitor* larvae and kept in laboratory conditions (24 ± 1 °C and 55–75% relative humidity), in ventilated boxes, partially filled with soil and dried wheat straw.

**Figure 2:**
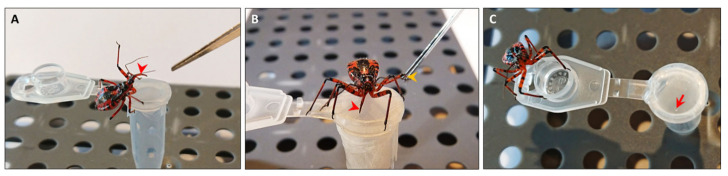
*Rhynocoris iracundus* venom collection. (**A**) To stimulate defensive venom production, *R. iracundus* was tapped using forceps causing it to display a defense posture and to raise its proboscis. (**B**) Then, the assassin bug was gently pinched on a rear leg (yellow arrowhead) with forceps causing it to use its proboscis (red arrowhead) to stab through the Parafilm stretched over a microcentrifuge tube. (**C**) Small venom drop released by the assassin bug on the Parafilm (red arrow).

We used the recently established method to stimulate *R. iracundus* nymphs to display a defense posture and subsequently collected the venom as illustrated in Figure 2 [30]. This procedure performed with one individual assassin bug refers to one venom harvesting event.

For cytotoxicity and antibacterial assays, the venom was collected from four individuals in four separate microcentrifuge tubes containing 100 µL phosphate buffered saline (PBS) each. The venom harvesting event was repeated ten times for each individual with 2–3 day intervals. The content of all tubes was pooled to obtain a venom stock solution.

The venom concentration was directly determined with PBS diluted venom by measuring the total protein concentration using the Pierce BCA protein assay kit (Thermo Scientific, Frankfurt, Germany). The measured protein concentration was 3470 µg/mL, which was used to determine the required venom concentrations for cytotoxicity and antibacterial assays.

For in vivo injections in *Galleria mellonella*, the venom harvesting event was repeated with four individuals in a total of either eight or 21 harvesting events, which corresponds to the low and high venom doses, respectively, used in subsequent experiments. Each microcentrifuge tube contained 75 µL of PBS.

For proteome analysis, the venom was collected from a total of eight venom harvesting events from four individuals, in one microcentrifuge tube without PBS.

All venom harvesting events were performed with 2–3 day intervals. After each venom harvesting event, the microcentrifuge tubes were briefly centrifuged and kept in −20 °C until further use.

### 2.2. Cell Lines and Cell Viability Assay

The cytotoxic effects of *R. iracundus* venom was tested against 2D cell cultures using two cancerous cell lines (Hepa 1–6 mouse hepatoma and Neuro 2a mouse neuroblastoma), non-cancerous cells (C2C12 mouse myoblasts), and Schneider 2 cells (S2 *Drosophila melanogaster* cell line). The first three cell lines were maintained in Dulbecco’s Modified Eagle’s Medium (DMEM) (Thermo Fisher Scientific, Frankfurt, Germany) supplemented with 4.5 g/L glucose, 110 mg/L sodium pyruvate and 584 mg/L L-glutamine, and 10% heat-inactivated fetal bovine serum (FBS). Incubation was at 37 °C in a humidified atmosphere with 5% CO_2_. S2 cells were cultured in Schneider’s medium (Thermo Fisher Scientific, Frankfurt, Germany), supplemented with 10% heat inactivated FBS, 2 mM L-glutamine, penicillin (100 U/mL) and streptomycin (100 µg/mL). Incubation was at 28 °C. Sub-culture was performed every 2–3 days, as soon as a confluency of 80–90% was reached.

The cytotoxic effects of assassin bug venom on the cell lines were evaluated using the resazurin-based alamarBlue assay (BioRad, Puchheim, Germany), which measures the cell viability after exposure to venom. Cells were seeded at a density of 2 × 10^4^ cells/well for C2C12 cells, 8 × 10^4^ cells/well for Hepa 1–6, and 1 × 10^5^ cells/well for Neuro 2a in 96-well, and 1 × 10^7^ cells/well for S2 cells. Incubation was for 48 h for the first three cell lines, with S2 cells incubated for 24 h. The cells were then exposed to assassin bug venom by incubating them for 4 h with final assassin bug venom concentrations of 43 µg/mL, 79 µg/mL, 174 µg/mL, and 848 µg/mL for the three cells and 174 µg/mL for S2 cells. After incubation, venom was removed from the cells by rinsing with PBS. 10% *v/v* alamarBlue reagent was added to each well and the cells were incubated for 1.5 h at 37 °C for C2C12, Neuro 2a and Hepa 1–6 cells, and for 1.5 h at 28 °C for S2 cells). The fluorescence of alamarBlue of the alive cells was measured in a Synergy H4 Hybrid Multi-Mode plate reader (BioTek Instruments, Vermont, United States) using excitation (528 nm) and emission (590 nm) filters. Cells incubated for the same period without venom (0 µg/mL venom) were used as negative controls (reference). The fitness of the control cells was verified by observing their shape using an inverted microscope Motic AE21 (Motic, Wetzlar, Germany). Respective media without cells were used as a blank. Two independent experiments were carried out for each concentration and performed in duplicates. The cell viability was calculated considering the reference as 100% viable cells and using Equation (1):

(1) $$\mathrm{Cell}\;\mathrm{viability}\;\left(\%\right)=\frac{\mathrm{Cell}\;\mathrm{count}\;\mathrm{venom}}{\mathrm{Cell}\;\mathrm{count}\;\mathrm{reference}}\times 100$$

For each cell line, statistical difference of cell viability between different concentrations of venom and the reference was evaluated using one-way ANOVA, with Dunnett’s multiple comparison test in GraphPad 5 Prism program (GraphPad Software Inc., United States). Differences were considered significant when *p* 0.05.

### 2.3. Antibacterial Assay

The effect of *R. iracundus* venom on bacterial growth was assessed using the Gram-positive bacterium *Listeria grayi* (DSM 20601), as well as the Gram-negative bacteria *Pseudomonas aeruginosa* (DSM 50071) and *Escherichia coli* (D31). Each strain was cultured overnight at 37 °C in a bacterial culture tube with 10 mL volume containing brain heart infusion broth (BHIB) liquid medium (Sigma-Aldrich, Darmstadt, Germany) for *L. grayi*, and lysogeny broth (LB) liquid medium (Sigma-Aldrich, Darmstadt, Germany) for *P. aeruginosa* and *E. coli*. Each bacterial suspension was then diluted in the respective media to reach an optical density at 600 nm (OD_600_) of ~0.005. Venom stock solution was added to the bacterial suspension at final venom concentrations of 43 µg/mL, and 174 µg/mL. The subsequent incubation was performed for 16 h at 37 °C and the antibacterial activity was determined as previously reported [31]. Negative control cultures without venom (0 µg/mL) containing medium and bacteria only were also included. The assays were carried out twice with comparable results.

### 2.4. Injection of Venom in Galleria Mellonella

*G. mellonella* larvae at their sixth larval instar were obtained alive from a local pet shop (Fauna Topics Zoobedarf Zucht und Handels GmbH, Marbach am Neckar, Germany) and were maintained as previously described [32]. We used larval and fresh pupal stages for all in vivo injections.

The venom from the microcentrifuge tubes with 21 or 8 venom harvesting events (see Section 2.1) was directly used by injecting 5 µL of diluted venom into individual *G. mellonella* larvae or pupae. Injection was performed subcutaneously in the thorax region using Hamilton micro-syringes and 22 gauge needles. Pupae and larvae which received a single 5 µL PBS injection instead of venom, and also untreated pupae and larvae which did not receive any injection, were used as controls. The effect of the venom on the larvae and pupae was assessed by evaluating melanization on their bodies’ surfaces 1 h, 4 h, and 24 h post injection. Paralysis was analyzed by observing their movements after a stimulation with tweezers 1 min, 30 min, 1 h, 4 h, 24 h after injection. *R. iracundus* releases varying quantities of venom in very small amounts, which does not allow precise quantification. Therefore, the venom dose injected in *G. mellonella* was calculated independently from the released amounts, as indicated in Equation (2):

(2) $$\mathrm{Venom}\;\mathrm{dose}\;\left(\%\right)=\;\frac{\mathrm{Venom}\;\mathrm{harvesting}\;\mathrm{event}\;\mathrm{count}\;}{V_{\mathrm{harvested}\;\mathrm{venom}\;+\;\mathrm{PBS}}}\;\times \;V_{\mathrm{injection}}$$

with: venom harvesting event count: 8 or 21, V_harvested venom+PBS_ = 75 µL, and V_injection_ = 5 µL.

### 2.5. Venom Gland Transcriptome Analysis

#### 2.5.1. RNA Isolation, Library Preparation and Illumina Sequencing

To prepare the samples for transcriptome analysis the assassin bugs were anaesthetized at −20 °C for 5 min, followed by dissection of the venom glands in pre-chilled (+4 °C) PBS. The dissected material was placed in tubes containing 500 µL of TRIzol (Merck KGaA, Darmstadt, Germany) and stored at −20 °C until RNA extraction. Total RNA was isolated from pooled venom glands (VG), body residues (BO), and gut (GU) using TRIzol according to the manufacturer’s instructions, followed by DNase treatment (Turbo DNase, Thermo Fisher Scientific, Schwerte, Germany) and further purification using RNA Clean and Concentrator 5 (Zymo Research, Irvine, United States). RNA quantity was determined using an Implen Nanophotometer and integrity of all RNA samples was verified using an Agilent 2100 Bioanalyzer and an RNA 6000 Nano Kit (Agilent Technologies, Palo Alto, United States). Transcriptome sequencing was carried out by GATC Biotech on an Illumina HiSeq3000 Genome Analyzer platform. Poly-A containing mRNAs were isolated from 1 µg total RNA using oligo-dT attached magnetic beads. The obtained mRNA was fragmented to an average of 260 bp and sequencing libraries were generated using the TruSeq RNA library preparation kit. Paired-end (2 × 150 bp) read technology was used for sequencing the *R. iracundus* samples, and resulted in a total of 24 million reads for the venom gland samples. Quality control measures, including the filtering of high-quality reads based on the score provided in the fastq files, the removal of reads containing primer/adapter sequences, and the trimming of the read length were carried out using CLC Genomics Workbench v11 (Qiagen, Hilden, Germany; http://www.qiagenbioinformatics.com).

#### 2.5.2. Transcriptome Assembly, Annotation, and Venom Protein Prediction

The de novo transcriptome assembly was carried out using CLC Genomics Workbench v11 with standard settings and two additional CLC-based assemblies with different parameters and then selecting the presumed optimal consensus transcriptome, as described previously (Vogel et al., 2014). The resulting final de novo reference transcriptome assembly of *R. iracundus* contained 38,109 contigs for the venom gland RNAseq data, with an N50 contig size of 1448 bp and a maximum contig length of 26,864 bp. The de novo transcriptome assembly of the combined RNAseq datasets contained 67,588 contigs, with an N50 contig size of 1169 bp and a maximum contig length of 25,112 bp. The transcriptomes were annotated using BLAST, Gene Ontology and InterProScan with Blast2GO Pro version 5.2 [33]. For BLASTx searches against the nonredundant NCBI protein database (NR database; accessed on 13 November 2019), up to 20 best NR hits per transcript were retained, with an E-value cutoff of ≤10^−1^ and a minimum match length of 15 amino acids to obtain the best homolog for the predicted short polypeptides. To assess transcriptome completeness, we performed a BUSCO (Benchmarking Universal Single-Copy Orthologs; http://busco.ezlab.org) analysis by comparing our assembled transcriptome against a set of highly conserved single-copy orthologs. This was accomplished using the BUSCO v3 pipeline [34], comparing the predicted proteins of the *R. iracundus* transcriptome to the predefined set of 1658 Insecta single-copy orthologs from the OrthoDB v9.1 database. This resulted in 89.5% complete and 4.9% missing BUSCO genes for the combined transcriptome assembly. Digital gene expression analysis was carried out using CLC Genomics workbench v11 to generate binary alignment mapping (BAM) files, and finally by counting the sequences to estimate expression levels, using previously described parameters for read mapping and normalization [35]. Post annotation, contigs were manually curated and separated into three categories: (i) putative toxins (contigs with homology to sequences previously identified as pathogenic toxins), (ii) non-toxins (e.g., contigs matching sequences such as housekeeping genes), and (iii) unassigned (contigs where no matches were assigned or BLAST E-values 1 × 10^−5^). Putative toxin contigs were subsequently curated in MEGA 7 by trimming to protein encoding regions only, (ii) removing contigs containing mutations which would interfere with expression of the encoded putative toxin (e.g., stop codons, frameshifts) and (iii) merging of identical sequences.

### 2.6. Venom Proteome Analysis

#### 2.6.1. SDS-PAGE, Protein Digestion and LC–MS Analysis of *R. iracundus* Venom

Venom proteins were separated by sodium dodecylsulfate polyacrylamide gel electrophoresis (SDS-PAGE) on 4–12% Criterion™ XT gradient gels (BioRad, Kabelsketal, Germany) with XT MES running buffer. Before loading, samples were mixed with XT sample buffer and reducing agent, and heated for 5 min at 95 °C. The Gel was run for 80 min at 120 V and stained using Coomassie Brilliant blue R250 (Imperial Protein stain, Thermo Scientific, Frankfurt, Germany). Molecular weights (kDa) of separated venom proteins were assessed using a pre-stained (Novex Sharp Pre-Stained Protein Standard, Invitrogen, Schwerte, Germany) and an unstained high mass precision protein marker (Broad Range Unstained Protein Standard, NEB, Frankfurt, Germany).

Two lanes of the SDS-PAGE gel were excised into 20 molecular weight fractions each, with the right lane containing twice the staining densities and twice the total protein amount compared to the left lane. Tryptic digestion was carried out as previously described [36].

For liquid chromatography-mass spectrometry (LC-MS) analysis, the extracted tryptic peptides were reconstituted in 30 µL aqueous 1% formic acid. Depending on staining intensity, 1 to 5 µL of sample were injected into the LC-MS/MS system consisting of an UPLC M-class system (Waters, Milford, United States) online coupled to a Synapt G2-si mass spectrometer (Waters, Milford, United States). Peptides were first on-line pre-concentrated and desalted using a UPLC M-Class Symmetry C18 trap column (100 Å, 180 µm × 20 mm, 5 µm particle size) at a flow rate of 15 µL min^−1^ (0.1% aqueous formic acid) and then eluted onto a ACQUITY UPLC HSS T3 analytical column (100Å, 75 µm × 200, 1.8 µm particle size) at a flow rate of 350 nL/min using following gradient: 3–10% B over 3 min, 10–20% B over 12 min, 20–30% B over 25 min, 30–70% B over 10 min, 70–95% B over 3 min, isocratic at 95% B for 1 min, and a return to 1%B (Buffers: A, 0.1% formic acid in water; B, 100% acetonitrile in 0.1% formic acid).

The eluted peptides were transferred into the mass spectrometer operated in V-mode with a resolving power of at least 20,000 full width at half height FWHM. All analyses were performed in a positive ESI mode. A 200 fmol/µL human Glu-Fibrinopeptide B in 0.1% formic acid/acetonitrile (1:1 *v/v*) was infused at a flow rate of 1 µL min^−1^ through the reference sprayer every 45 s to compensate for mass shifts in MS and MS/MS fragmentation mode.

Data were acquired using data-dependent acquisition (DDA) and data-independent acquisition (DIA, referred to as enhanced MS^E^). The acquisition cycle for DDA analysis consisted of a survey scan covering the range of *m/z* 400–2000 Da followed by MS/MS fragmentation of the 10 most intense precursor ions collected at 0.2 s intervals in the range of 50–2000 *m/z*. Dynamic exclusion was applied to minimize multiple fragmentations for the same precursor ions. LC-MS^E^ data were collected using alternating low energy (MS) and elevated energy (MSE) mode of acquisition over 0.5 s intervals in the range *m/z* 50–2000 with an interscan delay of 0.05 s. In low energy mode, data were collected at constant collision energy of 4 eV set on the trap T-wave device and ramped during scan from 20 to 45 eV in elevated MS^E^ mode. MS data were collected using MassLynx v4.1 software (Waters, Milford, United States).

#### 2.6.2. Data Processing and Protein Identification

DDA raw data were processed and searched against a subdatabase containing common contaminants (human keratins and trypsin) using ProteinLynx Global Server (PLGS) version 2.5.2 (Waters, Milford, United States). The following searching parameters were applied: fixed precursor ion mass tolerance of 15 ppm for survey peptide, fragment ion mass tolerance of 0.02 Da, estimated calibration error of 0.002 Da, one missed cleavage, fixed carbamidomethylation of cysteines, and possible oxidation of methionine. Spectra remained unmatched by database searching were interpreted de novo to yield peptide sequences and subjected for homology-based searching using MS BLAST program [37] installed on a local server.

MS BLAST searches were performed against *R. iracundus* sub-database obtained from in silico translation of *R. iracundus* transcriptome and against insecta databases downloaded from https://www.ncbi.nlm.nih.gov/, accessed on 3 March 2020.

In parallel, pkl-files of MS/MS spectra were generated and searched against *R. iracundus* subdatabase combined with NCBI nr database (https://www.ncbi.nlm.nih.gov/, accessed on 24 May 2020; containing 285,796,321 sequences) using MASCOT software version 2.6.2. The acquired continuum LC-MSE data were processed using ProteinLynx Global Server (PLGS) version 2.5.2 (Waters, Milford, United States). The thresholds for low/high energy scan ions and peptide intensity were set at 150, 30 and 750 counts, respectively. The processed data were searched against *R. iracundus* protein sub-database combined with Swissprot database downloaded from http://www.uniprot.org/, accessed on 13 July 2020. The database searching was performed at a false discovery rate (FDR) of 4%, following searching parameters were applied for the minimum numbers of: product ion matches per peptide (3), product ion matches per protein (7), peptide matches (1), and maximum number of missed tryptic cleavage sites (1). Searches were restricted to tryptic peptides with a fixed carbamidomethyl modification for Cys residues.

### 2.7. Protein Sequence Alignment

Multiple sequence alignments were performed using MAFFT 7.0 with default parameters and the E-INS-i refinement method [38,39]. The sequence logo was generated using WebLogo tool (Version 2.8.2) [40].

### 2.8. Phylogenetic Tree

To create the phylogenetic trees for Ptu1, hemolysin, and redulysin, we used the following approach: homologous sequences for each toxin family were obtained from Genbank. Sequences were aligned using MAFFT configured for highest accuracy [38] and non-aligned regions were removed with Gblocks (v 0.91b) [41]. The best-fit model was selected based on Akaike Information Criterion (AIC) and Bayesian Information Criterion (BIC) implemented in Molecular Evolutionary Genetics Analysis X (MEGA version X) [42]. MEGA was used for the following steps: obtaining the best tree topologies using the maximum likelihood (ML) method, estimation of the proportion of Gamma distributed sites (G), determination of the reliability of internal branches using the bootstrapping method (500 bootstrap replicates), and graphical representation and editing of the phylogenetic tree.

The percentage of trees in which the associated taxa clustered together is shown next to the branches. Initial tree(s) for the heuristic search were obtained automatically by applying Neighbor-Joining and BioNJ algorithms to a matrix of pairwise distances estimated using the JTT model (for redulysins and hemolysins), Le-Gascuel model (for Ptu1 family peptides) and then selecting the topology with superior log likelihood value. The tree is drawn to scale, with branch lengths corresponding to the number of substitutions per site. This analysis involved 25, 14, and 17 amino acid sequences for redulysins, hemolysins, and Ptu1 family peptides, respectively. All positions containing gaps and missing data were eliminated (complete deletion option). In the final dataset, there were a total of 112, 115, 21 positions for redulysins, hemolysins, and Ptu1 family peptides, respectively. The phylogenetic trees were inferred using MEGA X [42].

Species and accession numbers used in phylogenetic trees and alignments are listed in Supplementary Data S5.

## 3. Results

### 3.1. Venom Activity Against Mouse Cancer Cells and Healthy Mouse Cells

**Figure 3:**
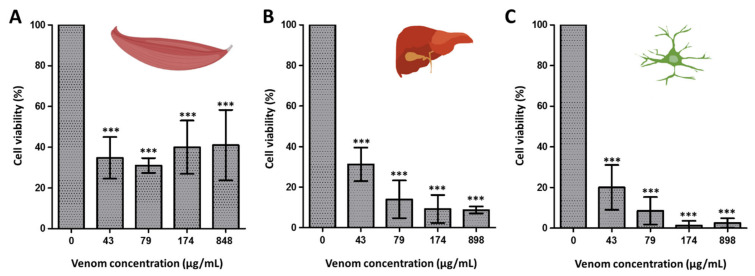
Cytotoxic effect of assassin bug venom. The effect of *Rhynocoris iracundus* venom was tested at different concentrations on healthy mouse myoblast cells C2C12 (**A**), on mouse hepatoma cells Hepa 1–6 (**B**), and on mouse neuroblastoma cells Neuro 2a (**C**). Shown are means and standard deviation values of the replicate samples. Statistical analysis was performed by one-way ANOVA with Tukey’s multiple comparison test applied for individual comparisons. Statistical significance of the differences of all venom concentrations against the control (0 µg/mL) are indicated with asterisks (*** *p* 0.0001).

To assess the activity of *R. iracundus* venom on different cell types, as well as its oncological potential, we performed viability assays with cancer cells, Hepa 1–6 (murine epithelial hepatoma cells), Neuro 2a (murine neuroblastoma cells) and C2C12 (healthy mouse myoblasts) using the resazurin-based alamarBlue assay. Exposure to the lowest venom concentration (43 µg/mL) showed a significant and potent cytotoxic effect in all tested cell lines compared to the control (Figure 3).

### 3.2. Venom Activity Against Bacteria

**Figure 4:**
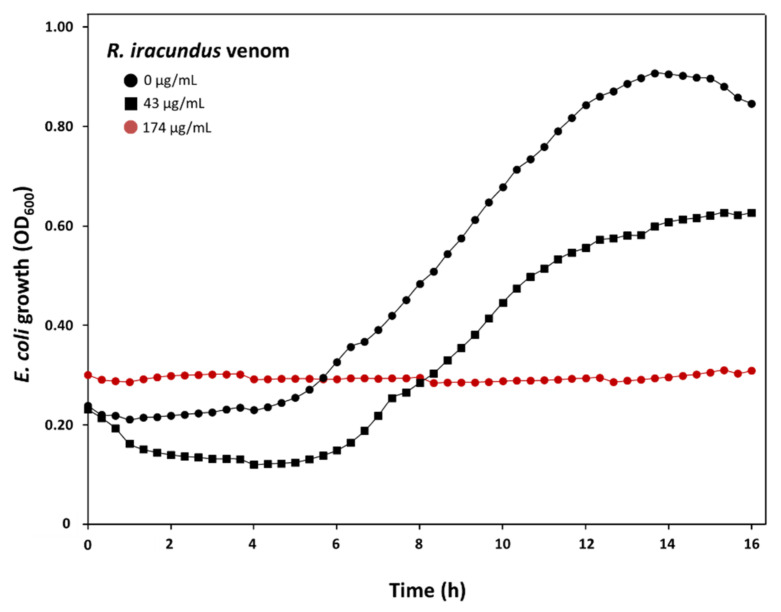
Effect of assassin bug *Rhynocoris iracundus* venom on *E. coli* D31 growth. Venom activity was tested using different concentrations of crude venom incubated together with *E. coli* D31. Bacterial growth was monitored by measuring OD_600_ values at 20 min intervals for 16 h. The effective venom concentration of 174 µg/mL is shown as a red line, and the bacterial growth curve at lower venom concentrations (43 µg/mL), or without venom (0 µg/mL), are displayed as black lines.

To evaluate the effect of *R. iracundus* venom against bacteria, we performed bacterial growth inhibition assays using Gram-positive *L. grayi* DSM 20601 and Gram-negative *P. aeruginosa* DSM 50071 and *E. coli* D31. No activity was observed on *L. grayi* and *P. aeruginosa* (Supplementary Figure S1). However, *E. coli* was susceptible to the highest venom concentration (174 µg/mL). A lower concentration of venom (43 µg/mL) delays the growth of *E. coli* (Figure 4).

### 3.3. Venom Activity Against Insect Cells and G. mellonella Pupae and Larvae

**Figure 5:**
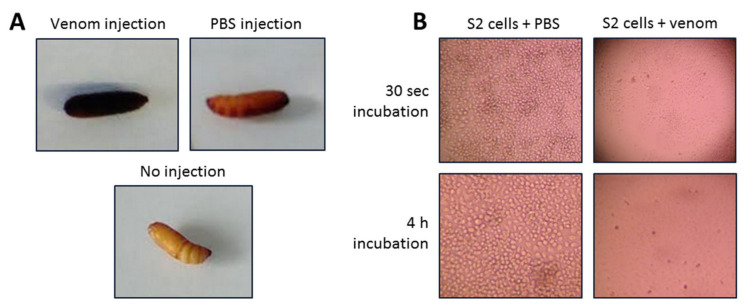
Effect of assassin bug *Rhynocoris iracundus* venom on insects. (**A**) Melanization of *G. mellonella* pupae in response to *R. iracundus* venom injection was assessed 4 h post injection. PBS injected and no injected pupae were used as controls. (**B**) Effect of venom on S2 cells after 30 s of co-incubation with venom. Morphological changes of S2 cells were monitored using an inverted microscope. Incubation of S2 cells together with 174 µg/mL venom for 30 s caused complete cell lysis. Incubation of S2 cells with PBS only (control) did not cause any visible cell lysis.

*R. iracundus* venom was tested for its cytotoxic effects on S2 cells using the resazurin based alamarBlue assay. Co-incubation of S2 cells with 174 µg/mL of venom strongly decreased the viability of S2 cells, causing 99% cell death during an incubation period of 4 h. S2 cells exposed to 174 µg/mL venom for as little as 30 s demonstrated morphological disruption by losing their shape, leading to lysis and cell death (Figure 5).

To investigate the effects of assassin bug venom on potential prey in vivo, we subcutaneously injected *R. iracundus* venom in *G. mellonella* larvae and pupae before evaluating the level of melanization and paralysis. Since the amount of venom during each envenomation can vary, we tested a low and a high dose of venom equivalent to 50% and 140% of the average venom dose, respectively (Table 1). For pupae and larvae, the first immobilization effects were already observed 1 min after post-injection. Strong melanization occurred after 4 h in larvae and pupae of *G. mellonella*. Three larvae were fully immobilized after 1 min, which progressively decreased after 4 h despite initiation of melanization. Furthermore, larvae became very soft 1 h post-injection. All tested *G. mellonella* pupae and larvae died, except one larva, which received only 50% of the average venom dose. This shows that the observed initial recovery in two out of three *G. mellonella* larvae was only temporary. Pupae and larvae, which were not injected remained healthy during the entire analysis and showed neither melanization responses nor a reduction in their mobility when stimulated with tweezers. PBS-injected pupae and larvae remained healthy as well but only slight and strictly localized melanization was observed at injection sites (Table 1).

### 3.4. Molecular Components of the Venom

#### 3.4.1. Overview of *R. iracundus* Venom-Gland Transcriptome

**Figure 6:**
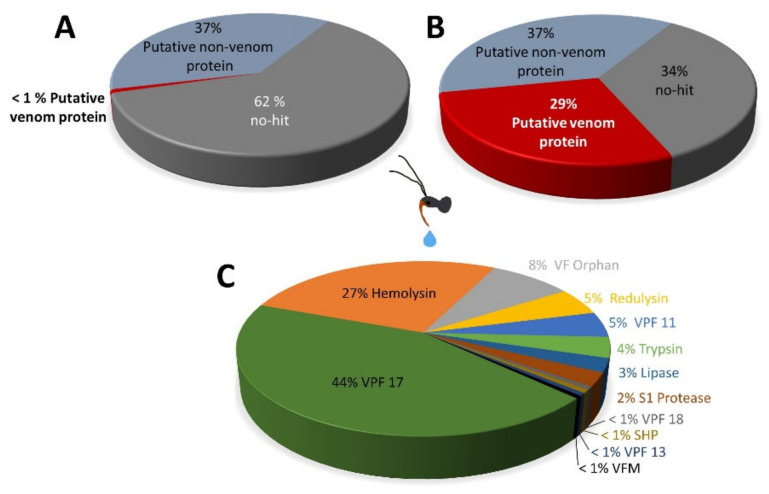
Types of transcripts found in *Rhynocoris iracundus* venom gland. (**A**) Relative proportion of venom protein encoding genes, non-venom protein encoding genes, and transcripts designated as “no-hit” (unknown). Venom protein genes account for 1% of the total transcripts (**B**) represents the relative transcript expression levels of the same categories. Among them, 29% of all sequence read counts of the transcripts are associated with the category “putative venom protein”. (**C**) Relative expression levels of toxin encoding genes only, displayed as % transcript expression of total putative venom protein encoding transcripts.

Sequencing of *R. iracundus* venom gland transcriptome yielded ~16 Mio trimmed, paired-end reads. The reads were subsequently assembled into 38,109 distinct contigs. Post-annotation, contigs were assigned into three categories: venom toxins, non-toxins and unassigned. The venom toxin transcripts accounted for 29% of total relative expression despite being comprised of only 0.1% of total transcripts (292 venom toxin transcripts). Notably, a substantial portion of the relative venom toxin expression was due to just two toxin families; venom family 17 (3 transcripts representing ~11% total expression) and hemolysins (4 transcripts representing ~7% total expression) (Figure 6).

Curation of the *R. iracundus* venom toxin transcripts revealed 35 individual venom toxin families (including the two mentioned above) represented by full length or partial transcripts. A total of 71 venom toxin transcripts were removed due to the presence of mutations which would render resulting proteins non-functional.

The majority (50%) of transcripts related to venom proteins belonged to the S1 protease (38 total, 10 full-length/28 fragment), trypsin (28, 15/33), orphan venom-family (25, 11/14), secreted hypothetical protein (14, 6/8), venom family 13 (11, 0/11) and chitinases (9, 3/6). However, the total relative expression of these families represented just 15% of total toxin transcript expression. In addition to venom family 17 and hemolysin already described above, other highly expressed toxin families include the orphan venom family (2% total expression), redulysin (~1%), and venom family 11 (~1%) (Figure 6).

Nevertheless, it should be noted that our transcriptomic approach identifies putative toxin precursor transcripts, and that our subsequent proteomic analysis validates whether the toxins were expressed.

#### 3.4.2. Venom Proteome of the Assassin Bug *R. iracundus*

**Figure 7:**
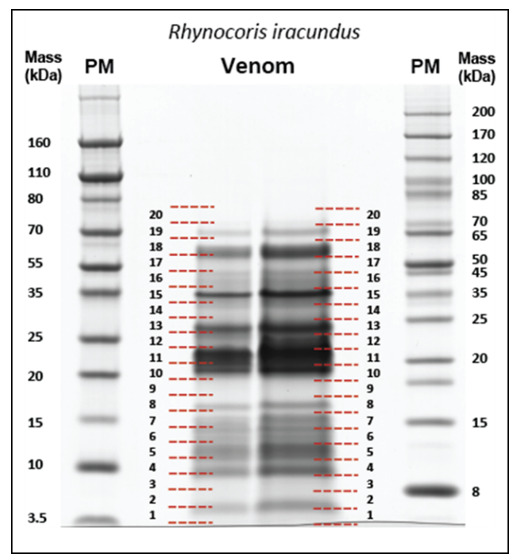
SDS-PAGE analysis of *Rhynocoris iracundus* venom proteins. Proteins collected from the venom glands of *R. iracundus* were separated by SDS-PAGE and stained with Coomassie Brilliant Blue R250. Numbers on the left and right of the lanes with venom indicate the 20 bands cut out from the gel and processed as individual samples for LC-MS/MS. PM = protein marker.

Based on the assembled venom transcriptome of *R. iracundus*, we broadly characterized the venom proteome by two sample runs using a standard bottom-up proteomics workflow. Venom samples were separated by SDS-PAGE (Figure 7) and both lanes were divided into 20 gel slices and subjected to in-gel digestion [43,44]. Resulting tryptic peptides were analyzed by peptide spectrum matching (PSM) based on the translated toxin sequences of the assembled transcriptome. The selected workflow does not allow any precise quantitative statements, but qualitative correlation can be achieved by identical protein matches.

**Figure 8:**
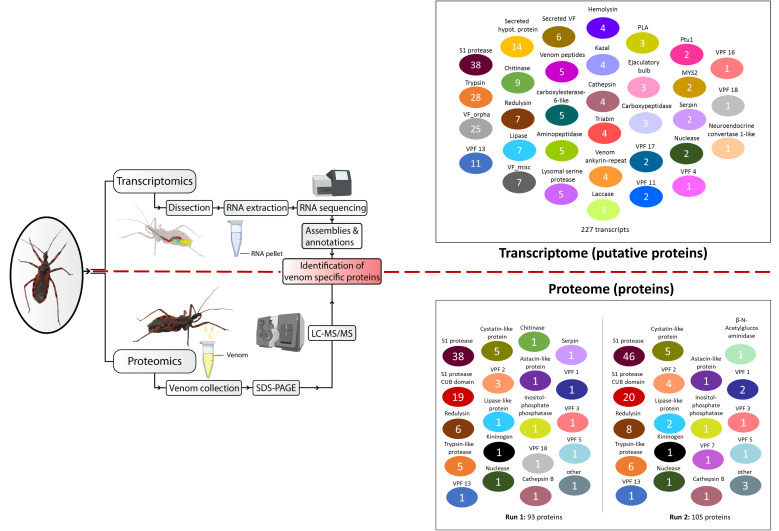
*Rhynocoris iracundus* transcriptome and proteome analysis workflow and identified venom protein families. In the venom gland transcriptome, 227 transcripts belonging to venom protein families were identified (**top right**), while venom proteome analysis resulted in the identification of 93 proteins and 105 proteins for each run, respectively (**bottom right**). Putative proteins and proteins belonging to the same protein family are indicated with identical colors.

In total, the proteomic analysis resulted in 93 and 105 protein matches for run 1 and run 2, respectively (Supplementary Table S1). Among the 35 individual toxin families identified in the *R. iracundus* venom proteome, six abundant and 12 low abundant toxin families were represented. A direct comparison of the venom composition for both sample runs showed a high agreement of identical toxins (87% and 67% of the total toxins in run 1 and run 2 respectively were detected in both sample runs) (Figure 8, Supplementary Table S1). The majority of the assassin bug venom proteins, identified by specific peptide fingerprints, belong to the proteolytic toxin families S1 proteases (27 isoforms), S1 proteases with CUB domain (16 isoforms), and trypsin-like proteases. Further, toxins belonging to minor diverse toxin families were identified, such as a venom protein family (10 isoforms) whose function is still uncertain, cytolytic toxin redulysin (6 isoforms), and cystatin-like proteins (5 isoforms) which are protease inhibitors. The remaining constituents of very low quantity include astacin-like protein, cathepsin B, inositol phosphatase-like proteins, nuclease-like proteins, venom protein kinase, venom phosphatase, and acetylglucosaminidase (Figure 8). Additionally, we found venom components in low quantity, such as chitinase-like protein, CUB domain protein, and serpin but also a number of unspecific proteins and contaminants for the first run (Supplementary Table S1).

A qualitative assessment of the assassin bug (*Rhynocoris iracundus*; Rirac) venom proteome in comparison to the predaceous Australian assassin bug (*Pristhesancus plagipennis*; Pplag) revealed strong similarities regarding the existence and relative proportion of the different toxin families [26]. Both analyses show a complex venom pool containing at least 127 (Pplag) or 110 (Rirac) toxin components, with the vast majority consisting of proteolytic proteins such as S1 proteases, with or without CUB domain (Pplag 65 proteins/Rirac 68 proteins). The cytolytic-active redulysin (Pplag 9/Rirac 8) venom protein family proteins (Pplag 19/Rirac 10), and cystatins (Pplag 7/Rirac 5) are also abundant toxin families in both venom proteomes. In addition, other minor abundant enzymes are present in both venoms.

### 3.5. Selected Toxins with Potential Cytotoxic and Antibacterial Activities

#### 3.5.1. Redulysins

**Figure 9:**
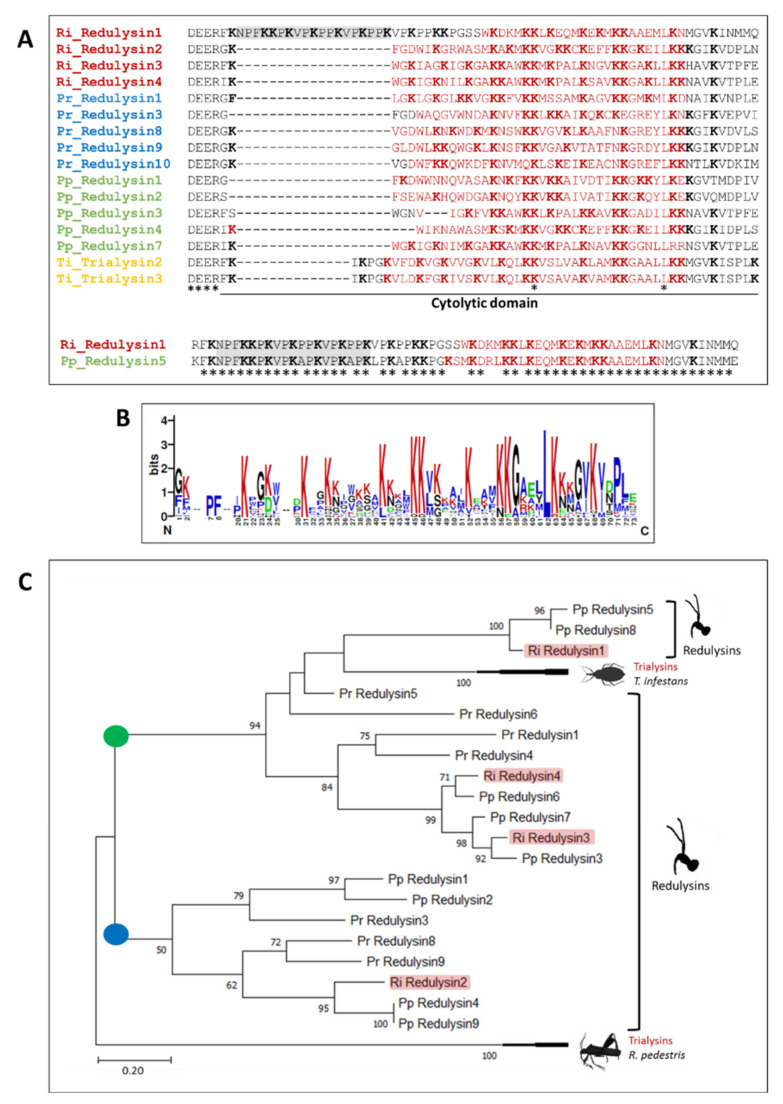
Redulyin catalytic domain characterization. (**A**) Cytolytic domain of redulysins from *R. iracundus* (Ri_Redulysin), *P. rhadamanthus* (Pr_Redulysin), *P. plagipennis* redulysin (Pp_Redulysin), and homolog trialysins from *T. infestans* (Ti_Trialysin). Coiled regions are highlighted in red. All Lys (K) residues are shown in bold and conserved amino acids are indicated with asterisks. Ri_Redulysin1 and Pp_Redulysin5 are aligned separately to show the high sequence similarity and the insertion in the cytolytic domain highlighted in gray. (**B**) WebLogo of the cytolytic domain alignment is represented by a stack of letters. The height of each letter indicates its frequency at that position of the sequence. The height of the overall stack is proportional to the sequence conservation. (**C**) Phylogenetic analysis of redulysins. Roots of the two main clades were highlighted in green and blue. The phylogenetic tree of all sequences mentioned above, as well as trialysins from *Riptortus pedestris* (Hemiptera: *Alydidae*) was inferred by using the Maximum Likelihood method and the Le_Gascuel_2008 model [46]. The tree with the highest log likelihood (−3253.56) is represented.

It has been previously demonstrated that redulysins are homologs of trialysins, which are Lys-rich cytolytic toxins described in the blood-feeding kissing bug *Triatoma infestans* [45]. The necessity of Lys (K) residues for cytolytic activity in the cytolytic region between Gly6 and Val32 of trialysin (AAL82381.1) was proven experimentally [45]. We compared the cytolytic domain of *R. iracundus* redulysins identified in our transcriptome analysis together with redulysins from the African assassin bug *Platymeris rhadamanthus*, the Australian assassin bug *P. plagipennis*, and trialysins from *T. infestans* (Figure 9A). The cytolytic domains are preceded by a conserved Asp (D)–Glu (E)–Glu (E)–Arg (R) sequence (Figure 9A). When comparing all redulysin sequences, only one of the *R. iracundus* redulysins (Ri_Redulysin1) have additional amino acids at the beginning of the cytolytic region. These additional amino acids are also found in one of the redulysins from *P. plagipennis* (Pp_Redulysin5) (Figure 9A). The last four residues of these additional amino acids align well with trialysins and show three out of four conserved residues. However, based on these data, it is not possible to state whether additional amino acids correspond to an insertion or a deletion in the rest of the sequences where additional amino acids were not detected (Figure 9A).

In the generated sequence logo, the overall height of each stack indicates the sequence conservation at that position (measured in bits) (Figure 9B). In the cytolytic region of redulysins and trialysins, Lys (K) residues are frequent and highly conserved. One Leu (L) is conserved in all sequences. We also aligned the complete sequences of the mentioned redulysins and trialysins and found that the C-terminal domain is stabilized by a pattern of eight conserved Cys (C) residues (Supplementary Data S1). To phylogenetically characterize *R. iracundus* redulysins, we used the same redulysin and trialysin sequences mentioned earlier together with trialysins from the broad-headed bug *Riptortus pedestris* (Hemiptera: *Alydidae*) (Figure 9C). The resulting phylogenetic tree is rooted with trialysins from *R. pedestris*. All redulysins and trialysins from *Reduviidae* fall into two main clades (roots highlighted in green and blue). Both of them display a clade expansion and have sequences from each assassin bug species. *R. iracundus* redulysins are more closely related to the redulysins from *P. plagipennis* than the redulysins from *P. rhadamanthus*.

#### 3.5.2. Kininogens

**Figure 10:**
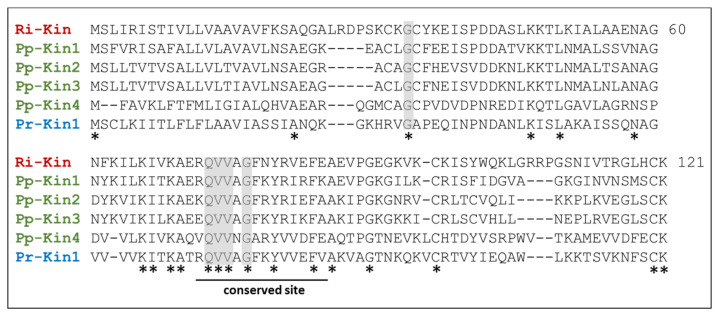
Sequence alignment of assassin bug kininogens. Kininogen sequences from *R. iracundus*, *P. plagipennis,* and *P. rhadamanthus* were aligned together. The conserved proteinase inhibitor cystatin site (Arg 72–Glu 85, Interproscan, IPR018073) is indicated with a line. Putative protease inhibition sites are highlighted in gray. Conserved amino acids are shown with asterisks (*).

In the *R. iracundus* proteome, we found a kininogen which belongs to the cystatin family. Kininogens are also found in *P. plagipennis* and *P. rhadamanthus*. We aligned the kininogen from *R. iracundus*, *P. plagipennis,* and *P. rhadamanthus* for comparison (Supplementary Data S2) and we found that, although the sequences align well, there are only 17% identical amino acids (Figure 10). We also found a conserved proteinase inhibitor cystatin site between Arg 72 and Glu 85, with 43% conserved amino acids. Four out of five putative protease inhibition sites are located within the proteinase inhibitor cystatin site.

#### 3.5.3. Chitinases

**Figure 11:**
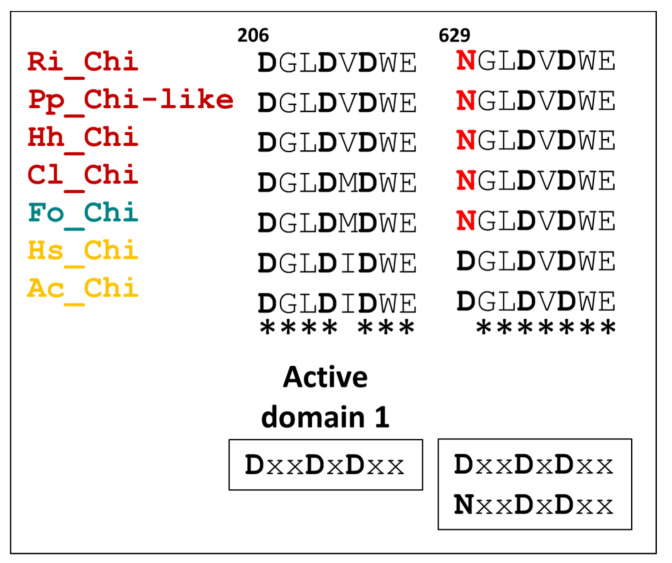
Alignment of chitinases. Chitinase and chitinase-like protein sequences from order Hemiptera (red, *R. iracundus*, *P. plagipennis*, *Halyomorpha halys*, *Cimex lectularius*), Thysanoptera (yellow, *Frankliniella occidentalis*) and order Hymenoptera (green, *Harpegnathos saltator* and *Apis cerana*) aligned and two active regions were found using interproscan website using *R. iracundus* chitinase protein sequence. Conserved residues from the active site motifs known for the activity of glycosyl hydrolase family 18 (GH18) indicated with bold. * indicates conserved amino acid residues.

In the *R. iracundus* proteome, we identified one chitinase. Based on the previously reported active domain motif for chitinases DxxDxDxE [47], we performed a motif search and aligned the identified active domain motifs from *R. iracundus* chitinase together with the chitinase-like protein from *P. plagipennis* (assassin bug) and the chitinases from *Halyomorpha halys* (stink bug), *Cimex lectularius* (bed bug), *Frankliniella occidentalis* (thrips), *Harpegnathos saltator* (ant), and *Apis cerana* (honey bee) (Figure 11). We found two conserved active domain motives D_206_xxD_209_xD_211_xE_213_ (active domain 1) and N_629_/D_629_xxD_632_xD_634_xE_636_ (active domain 2) (Figure 11, and Supplementary Data S3). To find out if other insect orders share Asn 629 (N_629_), which was found in the active domain 2 of assassin bug chitinases, we aligned 86 chitinases from eight insect orders (Supplementary Table S2). Our findings revealed that assassin bugs share N_629_ in the active domain 2 together with all insect orders, except for hymenopterans. The latter are the only representatives having Asp 629 (D_629_) in active domain 2.

#### 3.5.4. Hemolysins

**Figure 12:**
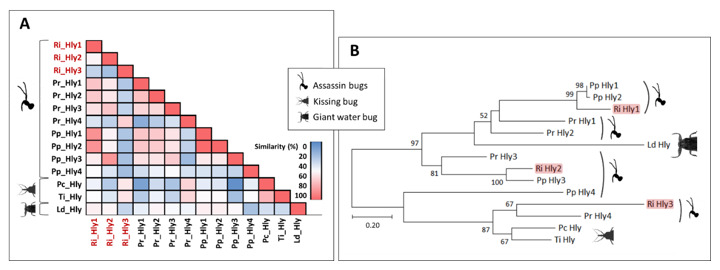
Characterization of hemolysins. (**A**) Pair-wise identity matrix of hemolysin sequences from insect order Hemiptera. Shown are hemolysins from the assassin bug *R. iracundus* (Ri_Hly), *P. plagipennis* (Pp_Hly), *P. rhadamanthus* (Pr_Hly), two kissing bugs *T. infestans* (Ti_Hly), and *Panstrongylus chinai* (Pc_Hly), and the giant water bug *Lethocerus distinctifemur* (Ld_Hly). (**B**) Phylogenetic tree of hemolysins. Hemolysin sequences from three assassin bugs *R. iracundus* (Ri_Hly) (highlighted in red), *P. plagipennis* (Pp_Hly), and *P. rhadamanthus* (Pr_Hly), two kissing bugs *T. infestans* (Ti_Hly), and *Panstrongylus chinai* (Pc_Hly), the giant water bug *Lethocerus distinctifemur* (Ld_Hly) were analyzed together. The phylogenetic tree was inferred by using the Maximum Likelihood method and Whelan and Goldman model (Whelan and Goldman, 2001). The tree with the highest log likelihood (−2664.67) is shown.

Three full length hemolysin transcripts (Ri_Hly_1, Ri_Hly_2, and Ri_Hly_3) were identified in the *R. iracundus* transcriptome. We used pairwise identity matrix of hemolysin sequences from various Hemiptera, including the assassin bugs *R. iracundus* (Ri_Hly), *P. plagipennis* (Pp_Hly), and *P. rhadamanthus* (Pr_Hly), both kissing bugs *T. infestans* (Ti_Hly) and *Panstrongylus chinai* (Pc_Hly), the giant water bug *Lethocerus distinctifemur* (Ld_Hly) and the bacteria *E. coli* (Ec_Hly) together to assess their similarity. The analysis suggested that hemolysin from *E. coli* has a maximal identity of 18% to insect hemolysins. *R. iracundus* Ri-Hly1 showed ~79% and ~78% identity to *P. plagipennis* hemolysins Pp_Hly1 and Pp_Hly2, respectively. *R. iracundus* Ri_Hly2 presented 75.35% identity to *P. plagipennis* Pp_Hly3 (Figure 12A). We also build a phylogenetic tree using hemolysins from Hemiptera. Within insect hemolysins, there are two sub-clades of hemolysins; the first clade consists of assassin bugs including two sequences from *R. iracundus* and the giant water bug hemolysin, whereas the second one includes kissing bug and assassin bug hemolysins with one sequence from *R. iracundus* (Figure 12B).

#### 3.5.5. Ptu1 Family Peptides

**Figure 13:**
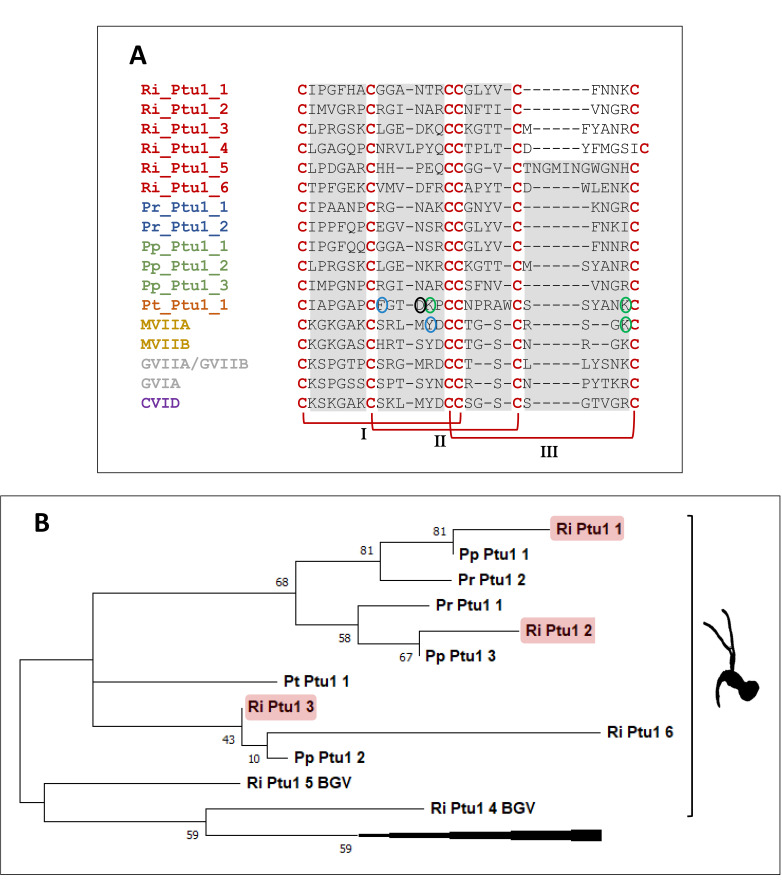
Characterization of Ptu1 family peptides. (**A**) Alignment of Ptu1 family peptides from *Rhynocoris iracundus* (Ri_Ptu1_V1, Ri_Ptu1_V2, (Ri_Ptu1_BGV3-Ri_Ptu1_BGV6), *P. rhadamanthus* (Pr_Ptu1_1 and Pr_Ptu1_2), *P. plagipennis* (Pp_Ptu1_1, Pp_Ptu1_2, Pp_Ptu1_3), *P. turpis* (Pt_Ptu1_1), and their homolog ω-conotoxins (MVIIA, MVIIB, GVIIA/GVIIB, CVID) from cone snails. The structure of Ptu1 family peptides includes three disulfide bridges (I, II, III) connecting six conserved cysteines, which are separated by four inter-cysteine loops. Residues playing a role in surface interaction such as aromatic residues (blue circle), acidic residues (black circle), and basic residues (green circle) are highlighted as published for Pt_Ptu1 and MVIIA. (**B**) Phylogenetic tree of Ptu1 family peptides. Ptu1 and ω-conotoxins mentioned above were used to generate a phylogenetic tree which was inferred by using the Maximum Likelihood method and Le-Gascuel model [46]. The tree with the highest log likelihood (−543.24) is presented. The cone snail image was created with Biorender.com.

Ptu1 is a N-type calcium channel blocker [48] neurotoxin [26]. In our transcriptomics analysis, we identified two complete Ptu1 family peptides in the venom gland assembly (Ri_Ptu1_1 and Ri_Ptu1_2) and four complete sequences in the combined assembly (Ri_Ptu1_3-Ri_Ptu1_6). We aligned the regions containing the knotting scaffold of Cys residues [48] from Ptu1 family peptides from *R. iracundus* and other species of assassin bugs (*P. rhadamanthus*, *P. plagipennis*, *Peirates turpis*) and from ω-conotoxins, which are Ptu1 homologues [48], from the cone snails *Conus magus* and *Conus moncuri* [49,50] (Figure 13A). We found that the Cys residues are highly conserved in *R. iracundus* Ptu1 and present the typical inhibitor cystine knot (ICK) scaffold. However, all inter-cysteine loops show sequence diversities (Figure 13A). In the well characterized toxins Pt_Ptu1 and MVIIA, we highlighted the residues in loop 2 which play a critical role in surface interactions [48]. Those residues are found in the same positions in Ptu1 family peptides from *R. iracundus* (Ri_Ptu1_3 and Ri_Ptu1_4) (Figure 13A). However, for the remainder of Ptu1, amino acids with different properties were found. The sequence similarities between Ptu1 family peptides from *R. iracundus* and the previously characterized Pt_Ptu1_1 and MVIIA [48] were below 45%.

Using a pair-wise identity matrix we show that Ri_Ptu1_1 has 84% sequence simi-larity to Pp_Ptu1_1, and Ri_Ptu1_2 has 78% sequence similarity to Pp_Ptu1_2 (Supplementary Data S4). Interestingly, phylogenetic analysis revealed that *R. iracundus* Ri_Ptu1_4 and Ri_Ptu1_5 fall into a separate branch together with cone snail ω-conotoxins. The rest of the assassin bug Ptu1 family peptides form a separate clade (Figure 13B).

## 4. Discussion

### 4.1. Primary Functions of Reduviid Venom: Capturing and Feeding on Arthropod Prey

Due to their abundance, arthropods represent a valuable food source for predators such as assassin bugs. However, arthropods have evolved to protect themselves from predatory attacks through the ability to quickly escape [51], possession of hard exoskeletons [52], the use of stingers [53] and venom [54]. Assassin bugs have learned and evolved to cope with these defense strategies. After a slow approach, reduviids ambush their prey, pin it down with their front legs, and quickly bite through soft body parts with a specialized straw-like proboscis. *R. iracundus* often feeds on honeybees *Apis* sp. and for such dangerous prey, *R. iracundus* prepares its proboscis in advance by elevating it towards the prey and in a sudden movement stabbing the prey with the proboscis and immediately pinning it down with the front legs at the same time (personal observation). A cocktail of venom with paralyzing components is injected through their particularly long proboscis [25]. Quick paralysis protects assassin bugs from injuries potentially caused by counterattacks from their prey and enables them to secure it for feeding purposes [25]. Since reduviids do not possess chewing mouth parts, components of the venom with liquefying properties are essential for feeding on their prey [25].

Notably, assassin bugs are able to modulate the composition of their venom in a context-dependent manner, either for defense or feeding purposes [25]. In our study, venom was collected after treating *R. iracundus* to display a defense posture. To understand its effects on their natural prey, we tested the venom on insects and insect cells. The injection of *R. iracundus* venom caused quick and full paralysis of *G. mellonella* larvae and pupae indicating the potency of the venom against insects, which is in line with the full paralysis observed on their natural preys such as honeybees; likely, the induced paralysis is a result of muscular or nervous dysfunction [55]. The earliest description of assassin bug venom effect on prey showing its paralytic activity, was described in 1773 [56] “once stung, the fly died immediately, which indicates that the assassin bug (*Reduvius personatus*) probably delivers potent venom”. In 1961, the paralysis observed in assassin bug prey was attributed to the disruption of cell membranes [23]. However, as it was observed with *R. personatus* and for other assassin bug species [25,57], *R. iracundus* venom also caused potent, quick and generalized paralysis in the entire insect body. This is in accordance with previous suggestions [25,27,57] that paralysis is not the result of localized cell membrane disruption, but it is due to molecular components of the venom targeting nerve cells. The neurotoxic effects on prey could be attributed, probably among other factors, to the presence of Ptu1 [26]. However, the role of Ptu1 family peptidesis controversial because previous publications mentioned that Ptu1 family peptides are neurotoxins that blocks N-type voltage-sensitive calcium channels [26,48], while the toxicity of Ptu1 family peptides could not be verified in vivo [58]. Further studies are required to understand its effects in vivo. Ptu1 family peptides are part of the inhibitory cystin knot (ICK) family capable of blocking nerve conduction [26,48]. Here, we found six different Ptu1 family peptides in the venom gland transcriptome of *R. iracundus*. In the well characterized Pt_Ptu1 from the assassin bug *P. turpis* and in its homolog MVIIA from the cone snail *Conus magus*, the respective aromatic residues Phe13 and Tyr13, surrounded by basic residues in loop 2, are critical for the binding of the toxin to the channel [48]. Notably, we found two out of six Ptu1 family peptides from *R. iracundus* clade together with cone snail ω-conotoxins. We have also observed, at the amino acid level, that one sequence of Ptu1 family peptide from *R. iracundus* possesses two identical residues as MVIIA in the same positions in loop two, and another sequence of Ptu1 family peptides from *R. iracundus* possesses one identical residue as Pt_Ptu1_1 in the same position. These residues play critical roles in surface interaction [48]. The rest of the Ptu1 family peptides have different amino acid residues at these positions. Potential candidates for sodium channel surface interaction residues with identical properties were found in different inter-cysteine loops in *R. iracundus* Ptu1 family peptides. The residues present in the inter-cysteine loops of ICK toxins can be mutated without significantly affecting their 3D structure [59], but are known to have an impact on binding specificity [60]. The cone snail ω-conotoxins are used as analgesic drugs for severe and chronic pain [5,61] and due to their similarities, Ptu1 family peptides could be interesting candidates for pharmaceutical applications. In addition, assassin bug venom Ptu1 family peptides revealed high conservation between *R. iracundus* and the Australian *P. plagipennis* (Ri_Ptu1_1/Pp_Ptu1_1: 84% similarity, Ri_Ptu1_2/Pp_Ptu1_3: 78% similarity), which suggests that these species share a relatively recent common ancestor of Ptu1 family peptides in geographically distant assassin bug species. 

Assassin bug venom contains a high proportion of molecular components with cytolytic and enzymatic activities involved in tissue liquefaction [26]. In vivo injected *G. mellonella* larvae and pupae with *R. iracundus* venom lost their bodies’ rigidity and got soft to the touch. The addition of *R. iracundus* venom to S2 insect cells caused 99% cell lysis within 30 s, demonstrating the potency of *R. iracundus* venom to lyse and liquify their prey. In our transcriptome and proteome data we found digestive enzymes such as S1 proteases, S1 protease + CUB, and also chitinases which are, possibly among other molecular components, responsible for the observed insect softening and cell lysis [26,62].

In other zoophagous heteropterans, such as *P. plagipennis* chitinase-like proteins were also detected. Chitinases are also present in salivary glands of phytophagous hemipterans, e.g., *Oncopeltus fasciatus* [63] but were not found in hematophagus kissing bugs such as *Rhodnius prolixus* [64]. Despite the phytophagy of *O. fasciatus*, the presence of chitinases in its salivary glands is probably important due to its occasional cannibalistic behavior [65], which is rare in kissing bugs [66]. Insect chitinases have the primary role to digest chitin during metamorphosis [67,68]. Their presence in salivary glands and venom glands of insects further suggests their importance toward feeding and defense purposes. We noticed that chitinases possess two active domains: active domain 1 (DxxDxDxx) present in all insects, while active domain 2 presented a sequence specific to hymenopterans (DxxDxDxx), which is different from all other insect orders (NxxDxDxx). The sequence DxxDxDxx was described essential for the enzymes’ activity [47]. We suggest that in hymenopterans Asn (N(1)) mutated to Asp (D(1)) in the active domain 2. The impact of this mutation in chitinases is not known yet. However, what is known is that chitinases have multifunctional roles, e.g., regulation of inflammation or intratumoral processes, potentially making them attractive candidates for cancer therapy and immunomodulation [69].

Beside paralysis, strong melanization of *G. mellonella* was also observed; however, it appeared a few hours post injection. Melanization could be a secondary response of the cell lysis, which was characterized by softening of the *G. mellonella* bodies. Melanization plays a vital role in various physiological processes in insects, including wound healing and immunity [70].

### 4.2. Versatility of Assassin Bug Venom towards Defense Purposes

Using their proboscis, assassin bugs are capable of inflicting painful bites to humans, with local and systemic symptoms, as reported from *Zelus* sp. [29]. This shows that assassin bugs can use their proboscis to stab and deliver venom for self-defense purposes, and that the venom has potent bioactivity on potential threats, such as predators. For example, mice can be severely affected by assassin bug venom, even resulting in their death due to venom-inflicted respiratory paralysis [24].

When harassed, *R. iracundus* immediately takes a defense posture, lifting its front legs, and displaying its venom delivery organ. We noticed that the defense posture of assassin bugs resembles those typically observed in arachnids [71]. This enables assassin bugs to raise up towards predators of greater size and the position of their proboscis prepares them for a direct attack. Furthermore, a small drop of venom sticking to the end of the proboscis was often released in a fashion similar to disturbed funnel web spiders [72].

Assassin bug *Holotrichius innesi* venom causes quick respiratory paralysis and leads to death in mice [24] which indicates that the venom is also potent against their natural enemies such as mice and potentially birds. To understand the effects of assassin bug venom on mammalian tissues, we tested *R. iracundus* venom on various cell types. The cytotoxicity assays performed with *R. iracundus* venom demonstrated significant lysis of all tested cell types: hepatoma cells, murine neuroblastoma, as well as on murine myoblasts demonstrating its activity on diverse cells. The activity of *R. iracundus* venom on neuroblastoma is especially interesting in regards of the observed paralysis in mice. Paralysis due to assassin bug venom was also noticed in insect preys which was first explained by cell membrane breakdown [23]. However, considering the similarity with ω-conotoxins, and the presence of conserved ICK motifs, Ptu1 family peptides block N-type voltage-sensitive calcium channels, which suggests that their presence in assassin bug venom could possibly be associated with pain [73] and paralysis [48]. However, Ptu1 was tested against a range of vertebrates and invertebrates, including insects, and it did not show toxicity [58]. All in all, this suggests that Ptu1 could be responsible for the relief of pain or counteract paralysis. Fast paralysis observed in insects in our experiment, and in insects and vertebrates in other studies [23,24,25,56] as well as sensations of numbness and tingling in human [29] can be associated with other yet undescribed neurotoxins.

The lysis of murine neuroblastoma, as shown for the first time in our study, and the observed murine respiratory paralysis [24] suggest that both neurolysis and paralysis are caused by assassin bug venom. Generalized and rapid paralysis in assassin bug prey enables assassin bugs to protect themselves from injuries. We suggest that neurolysis counteracts paralysis locally, in the region of the assassin bugs’ sting, due to the destruction of neuronal networks and may serve for prey digestion and feeding purposes.

The observed cytotoxic effect against the tested cell types can be attributed, among other factors, to the presence of redulysins, hemolysins [74], and kininogen [75] which were found in our transcriptome data. Redulysins and kininogen were also found in the *R. iracundus* venom proteome.

Redulysins, homologs of trialysins [27] for which cytotoxic activity was demonstrated [45], were among the four protein families with highest relative expression levels. Alignment of the cytolytic domains of the four complete *R. iracundus* redulysins with published sequences from other assassin bug species and from kissing bugs revealed a high proportion of Lys residues in all redulysins, which is required for their cytotoxic activity [45]. The remaining amino acid residues found within the cytolytic domain can be responsible for varying degrees of cytotoxic activity against mammalian cells [45].

Notably, one of the *R. iracundus* redulysins (Ri_Redulysin1) aligned well with the redulysin from *P. plagipennis* (Pp_Redulysin5) and, when comparing both of them to all other redulysins and trialysins, they showed an additional Lys-richregion at the beginning of the cytolytic domain. Whether and how this insertion additional Lys-rich region affects the activity of the redulysins should be studied in more detail in regards of their cytolytic properties.

Hemolysins are known as pore-forming [76] exotoxins and are able to lyse erythrocytes [77]. One partial and three complete sequences were found in the *R. iracundus* venom transcriptome. Based on the pair-wise identity matrix and phylogenetic tree, *R. iracundus* hemolysins have sequence similarities with hemolysins from *P. plagipennis*.

In our previous study we already discovered that *R. iracundus* venom causes only ~6% of hemolysis on porcine erythrocytes [30] and erythrocytes from different vertebrate species show different levels of hemolysis [57]. This suggests that assassin bug venom has selectivity on different erythrocyte types making assassin bug venom interesting for leukemia studies.

Finally, our third candidate for cytotoxic activity are kininogens, which are a family of cysteine protease inhibitors. They have similarities to histidine-rich glycoproteins and cystatin-related proteins [78,79]. We aligned kininogens from the assassin bugs *R. iracundus*, *P. plagipennis*, and *P. rhadamanthus* and found a maximum sequence similarity (~58%) between the kininogens of *R. iracundus* Ri_Kin and *P. plagipennis* Pp_Kin2. Kininogen inhibits migration and invasion of cancer cells in vitro [80], and overexpression of the kininogen KNG1 was shown to decrease tumor growth and to promote the apoptosis of glioma cells [75]. The observed cytotoxic activity on cancer cells in our study could therefore, possibly among other candidates, be attributed to kininogens. Therefore, kininogensA from assassin bugs kininogens are interesting to study in more detail regarding their antitumor activity. Interestingly, assassin bug venom did not negatively affect collagen tissues [23] and molecular components with cytolytic activities could therefore be interesting to study in cancer of bones and joints.

### 4.3. Keeping the Glands Clean: The Antibacterial Activity of Assassin Bug Venom

In their natural environment, and especially during feeding, assassin bugs are exposed to various microorganisms. Insects can carry entomopathogens such as bacteria, fungi, and viruses [81,82] which could potentially affect and kill their predatory insects [83,84] via toxemia, bacteremia, or septicemia [85]. To analyze whether *R. iracundus* venom protects the assassin bug from potential pathogens, we tested its venom against Gram-positive and Gram-negative bacteria and found venom-mediated bacterial growth inhibition on Gram-negative *E. coli* only. The selective antibacterial effect against *E. coli* highlights the potential of *R. iracundus* venom to identify molecular compounds, which could be used to treat human diseases caused by *E. coli* or other Gram-negative bacteria. Therefore, we suggest that *R. iracundus* venom can prevent microbial colonization of the glands, protecting the assassin bug against pathogens. Due to their similarity to trialysins [27], redulysins represent potential candidates for the observed growth inhibition of bacteria. Trialysins were shown to have pore forming activities in lipid bilayers [45]. Therefore, the identified redulysins in our *R. iracundus* venom are interesting candidates to investigate in more detail in regards to their potential antibacterial properties.

## 5. Conclusions

The need for novel biomedical tools is ever increasing, thus driving researchers to continuously explore novel and untapped opportunities. In this context, some focus has fallen on animal venoms since the toxins they are constituted of perform specialized physiological functions within their prey or predators. The specificity as well as potency of animal venom toxins renders them highly interesting for biomedical research. However, whilst the venom cocktails of larger animals (e.g., snakes, spiders, and scorpions) have been investigated for decades, the ones from smaller animals remain poorly characterized. Only recently, assassin bugs and their multifunctional venom has entered the limelight. Therefore, in this study, we investigated the venom of the assassin bug *Rhynocoris iracundus* and discovered a diverse array of toxins and bioactivities. Indeed, we found that redulysins, kininogens, chitinases, hemolysins, and Ptu1 family peptides appear to be responsible for paralysis of arthropods and vertebrates, neurolysis and cytolysis of insect and mammalian cells, as well as insecticidal and antibacterial activities. Therefore, our study provides a promising basis for further investigation and characterization of specific *R. iracundus* toxins, and the candidate molecules presented here could, in future, serve as templates for novel biotherapeutics.

## Figures and Tables

**Table 1:** Effect of *Rhynocoris iracundus* venom on *Galleria mellonella*. *G. mellonella* larvae and pupae were injected subcutaneously with low (50%) and high (140%) venom doses. Paralysis and melanization observations were recorded from 1 min until 24 h post injection.

*Galleria mellonella* Stages	Venom Dose (%)	Incubation after Injection								Replicates
		1 min	30 min	1 h		4 h		24 h		
		Paralysis	Paralysis	Paralysis	Melanization (%)	Paralysis	Melanization (%)	Paralysis	Melanization (%)	
**Pupae**	140	none	n.a.	none	0	full	70	full	100	4
		full	n.a.	full	0	full	70	full	100	1
	50	full	n.a.	full	0	full	40	full	90	7
	PBS only	none	none	none	0	none	5	none	5	12
	No injection	none	none	none	0	none	0	none	0	12
**Larvae**	50	none	partial	n.a.	n.a.	full	60	full	100	5
		full	full	n.a.	n.a.	full	30	full	100	3
		full	full	n.a.	n.a.	partial	20	partial	100	1
		full	full	n.a.	n.a.	partial	20	full	100	1
		full	full	n.a.	n.a.	partial	20	n.a.	0	1
	PBS only	none	none	n.a.	n.a.	none	5	none	5	12
	No injection	none	none	n.a.	n.a.	none	0	none	0	12

n.a. = not applicable (paralysis and/or melanization were not assessed).

## Data Availability

The short read data described herein have been deposited in the EBI short read archive (SRA) with the following sample accession numbers: ERS6419927-ERS6419929. The complete study can also be accessed directly using the following URL: http://www.ebi.ac.uk/ena/data/view/PRJEB44908. Mass spectrometry proteomics data (.mgf and .raw files) have been deposited with the ProteomeXchange Consortium (http://proteomecentral.proteomexchange.org) via the MassIVE partner repository under project name “Eurasian assassin bug *Rhynocoris iracundus* venom proteome” with the data set identifier PXD 026055.

## Supplementary Materials

The following are available online at https://www.mdpi.com/article/10.3390/biomedicines9070819/s1, Figure S1: *Rhynocoris iracundus* venom against *Listeria grayi* and *Pseudomonas aeruginosa* growth, Table S1: Venom proteome information of *Rhynocoris iracundus*, Table S2: Chitinase active domain 2 (DxxDxDxx), motif information from insect order Hemiptera, Lepidoptera, Coleoptera, Thysanoptera, Dictyoptera, Phthiraptera, and Hymenoptera, Data S1: Amino acid sequence alignment of assassin bug redulysins together with homolog from kissing bug trialysins, Data S2: Pair-wise identity matrix of Kininogen sequences from the assassin bug *Rhynocoris iracundus*, Australian assassin bug *Pristhesancus plagipennis*, African assassin bug *Platymeris rhadamanthus*, Data S3: Alignment of Chitinases from order Hemiptera (assassin bugs, bed bugs, stink bugs), Thysanoptera (thrips) and order Hymenoptera (ant, bee), Data S4: Pair-wise identity matrix of Ptu1 family peptides of the assassin bug *Rhynocoris* *iracundus*, Australian assassin bug *Pristhesancus plagipennis*, African assassin bug *Platymeris rhadamanthus*, Data S5: Accession numbers of the sequences which are used for the alignments and the phylogenetic analyses.
