# Supplementary material for: Hexapod Assassins’ Potion: Venom Composition and Bioactivity from the Eurasian Assassin Bug Rhynocoris iracundus
Source: Biomedicines. 2021 Jul 14;9(7):819. doi: 10.3390/biomedicines9070819 (PMC8301361; doi:10.3390/biomedicines9070819)
Supplement: Supplementary file 1 [file biomedicines-09-00819-s001.zip › Supplementary Data S1- Redulysin Alignment.pdf]

```
Ri_Redulysin1     PKVPKPPKVPKPPKKPGSSWKDKMKKLKEQMKEKMKKAAEMLKNMGVKINMMQCE-EKTC
Ri_Redulysin2     ---GKFGDWIKGRWA---SMKAKMKKVGKKCKEFFKKGKEILKKKGIKVDPLNCN-GNVC
Ri_Redulysin3     ---FKWGKIAGKIGK---GAKKAWKKMKPALKNGVKKGAKLLKKHAVKVTPFECD-DKTC
Ri_Redulysin4     ---IKWGKIGKNILK---GAKKAWKKMKPALKSAVKKGAKLLKKNAVKVTPLECE-EKTC
Pr_Redulysin1     ---GFLGKLGKGLKK---VGKKFVKKMSSAMKAGVKKGMKMLKDNAIKVNPLECE-GKFC
Pr_Redulysin3     ----GFGDWAQGVWN---DAKNVFKKLKKAIKQKCKEGREYLKNKGFKVEPVICEGGKKC
Pr_Redulysin5     ---KGLKKFGKAVVQ---KGKKFIKKVKGPMKNIMKKGAALLKNLGVKINPLQCE-EKTC
Pr_Redulysin6     AE-LGLKKFGGQLAKGGKKFVKAMKKMKEPIKKLAKQGLEILKKFGVKIIPLHCE-GKTC
Pr_Redulysin7     SKNKGNKVTSKDPSKPKKKKKFSLKGIKESIKKWTKKGMELLKNAGVKVNPFECA-EKRC
Pr_Redulysin8     ---GKVGDWLKNKWD---KMKNSWKKVGVKLKAAFNKGREYLKKKGIKVDVLSCQ-GSKC
Pr_Redulysin9     ---GKGLDWLKKQWG---KLKNSFKKVGAKVTATFNKGRDYLKKKGIKVDPLNCQ-GNKC
Pr_Redulysin10    ---GKVGDWFKKQWK---DFKNVMQKLSKEIKEACNKGREFLKKNTLKVDKIMCN-EGTC
Pp_Redulysin1     ----GFKDWWNNQVA---SAKNKFKKVKKAIVDTIKKGKKYLKEKGVTMDPIVCEGGKQC
Pp_Redulysin2     ----SFSEWAKHQWD---GAKNQYKKVKKAIVATIKKGKQYLKEKGVQMDPLVCEGGNQC
Pp_Redulysin3     ---FSWGNV---IGK---FVKKAWKKLKPALKKAVKKGADILKKNAVKVTPFECD-DKTC
Pp_Redulysin4     ---IK---WIKNAWA---SMKSKMKKVGKKCKEFFKKGKEILKKKGIKIDPLSCT-GNTC
Pp_Redulysin5     PKVPKAPKLPKAPKKPGKSMKDRLKKLKEQMKEKMKKAAEMLKNMGVKINMMECE-EKTC
Pp_Redulysin6     ---INWGKIGKGILK---GAKKAWKKMKPALKSAVKKGAKLLKKNAIKVTPFECE-EKSC
Pp_Redulysin7     ---IKWGKIGKNIMK---GAKKAWKKMKPALKNAVKKGGNLLRRNSVKVTPLECD-DKTC
Pp_Redulysin8     PKQPKLPKPPKPPKKPGSSFKDKMKKLKDKMKEKMKKAAEMLKNMGVKINMMECE-EKTC
Pp_Redulysin9     ---IK---WIKNAWA---SMKSKMKKVGKKCKEFFKKGKEILKKKGIKIDPLSCT-GNTC
Ti_Trialysin1     ---IKPGKVLDKFGKIVGKVLKQLKKVSAVAKVAMKKGAALLKKMGVKISPLKCE-EKTC
Ti_Trialysin2     ---IKPGKVFDKVGKVVGKVLKQLKKVSLVAKLAMKKGAALLKKMGVKISPLKCE-EKTC
Ti_Trialysin3     ---IKPGKVLDKFGKIVSKVLKQLKKVSAVAKVAMKKGAALLKKMGVKISPLKCE-ENTC
Rp_Trialysin1     -----------AFWD---KAFNLLKKV-------------------LHVSPLTCN-GANC
Rp_Trialysin2     -----------AFWD---KLFNFFKKT-------------------LSVEPLNCQ-GANC
                                                                        *     *

Ri_Redulysin1     KTCISFSIPGFEKTYCISMRFMRTNKNTYVIFGIGKEGSPA------VEQKLKLG-DLPS
Ri_Redulysin2     KSCVDFTLKK--RKFCVEAKFQSTS----IQMSLTKQKDDQEPKAVIGPFTINIG-NVPT
Ri_Redulysin3     KTCINLVFLG--VSACLKATISREDKVTYFTIAGLVNDSPQ------FEEKIKLG-AVPR
Ri_Redulysin4     KTCIKLTFLG--MSACLRYTITRTNKNTYFTIAGEVNGNTQ------FEEKIRLG-DMPR
Pr_Redulysin1     KSCISML----TKTVCIKGTAERINKVAYILINPTIDGENK------GEIKLKVG-DVPR
Pr_Redulysin3     KSCIFLPSMK--KKFCLELTFYQENKIKFIKVACIRERDDKEPKEVFPPMNIALG-KPST
Pr_Redulysin5     KTCIIMKIPT-EKSFCLQMTIMRTNKASYLKIALTKDDVSK------FEQNIKLG-DVPR
Pr_Redulysin6     KSCIIFTIPA-EISLCVQVTFLRTNKATYLIIALTKDDATL------FEKRITMG-DVSN
Pr_Redulysin7     KSCIKINLGK-EYSACAHIRFLNTNKGTYIVWGIGTGDKAA------FEKKMKLG-SLTD
Pr_Redulysin8     RSCLIFTLKK--KQFCLEFEFSNTG----LKISLTKQKDDQEPKIMLGPFNIKTG-NVPQ
Pr_Redulysin9     RSCVIFTLKP--KKFCIEYVFSTSA----ITVSLIKEKDDEE-KVLLGPFTIKTG-NIPK
Pr_Redulysin10    KSCVKIPLMP---SVCFEGDLSLDEG---FKFSVYFFKDGKILNYIVSKYFVKYG-DTLK
Pp_Redulysin1     KTCLVFLKMK--KKFCMELKFFSKDKVKYINVKLIRERDDKEPKELLKPIDIPIS-KAST
Pp_Redulysin2     KTCLSFLKMK--KKFCMEVKFFSKGELRFANVKLIRERDDKEPKELFKPLDIPMG-KESN
Pp_Redulysin3     KTCINLVFLG--MSACMRATISREDKITYFTIAGEVNDSPQ------FEEKIKLG-DVPR
Pp_Redulysin4     KSCIDFTMKK--RKFCIEALFQSSA----IQVSLTKQKAEQEPKAIIGPFSINIG-DIPK
Pp_Redulysin5     RTCISFSLPGFERTYCISMRFMRTNKNTYVIFGMGLKDKPS------KEIKLTLG-NLPS
Pp_Redulysin6     KTCINLVFLG--LSACLKATITRTNKNTYFTISGEVNGNTQ------FEEKIRLG-DLPR
Pp_Redulysin7     KTCINLVFLG--LSACLKATISREDKVTYFTIAGDVNGSPQ------FQEKIKLG-DVPR
Pp_Redulysin8     KTCISFSLPGFERTYCISMRFMRTNKNTYVIFGMGLKDKPS------KEIKLTLG-NLPS
Pp_Redulysin9     KSCIDFTMKK--RKFCIEALFQSSA----IQVSLTKQKAEQEPKAIIGPFSINIG-DIPK
Ti_Trialysin1     KSCVIFKIPT-ENSFCLTIRFMKTNIATYLVVAGEINRKSK------FEEKLKLG-NMPR
Ti_Trialysin2     KSCVIFKIPT-ENSFCLTIRFMKTNIATYLVVAGEINRKSK------FEEKLKLG-NMPR
Ti_Trialysin3     KSCVIFKIPT-ENSFCLTIRFMKTNIATYLVVAGEINRKSK------FEEKLKLG-NMPR
Rp_Trialysin1     KTCLSA--FG--KGGCVTVNYNKQAGAN---AAIQVNVQSNSGRNI-WNGSINAGRPVPS
Rp_Trialysin2     RTCVKV--LG--KGGCIGIGYNKQGGAN---AAIQVSVQSGSGRNI-WNGNINAGRPVPS
                    *            *
```

```
Ri_Redulysin1     CFSLG-ELLGKVCVKCVEGRAKTSQGQGNVNFCLGLLAEKFSLGAKFCATYQDKK-LKIR
Ri_Redulysin2     CKSLG-KVIGPVCLQGVEGRAKSSKGQAHVNFCAAALLKNYGIGAKFCIGYVDGK-FTGS
Ri_Redulysin3     CVNAG-SVIGKICIKGMEGKGKSSQGKAQVNFCLGFLAEQHNIGVKVCATYENKK-FKIK
Ri_Redulysin4     CVNAG-SVIGKLCLKGLEGKGKSSQGKAQVNFCLGFLAEKHNVGVKVCASYE--------
Pr_Redulysin1     CVNFG-GLLGKICLKGLEGKAKSSSGQANVKFCLAMLAEKYNVGCKFCAIYANKK-FK-P
Pr_Redulysin3     CIKLG-SILGKLCLQGVEGKAKSSKGQANINFCLAFVLKNFGVGAKLCGSYEQGK-MNFK
Pr_Redulysin5     CLNLG-GFIGKICLKGIEGRAKSSQGQGNVNFCLGIVAEKFGFGCKFCATYENKK-LKVK
Pr_Redulysin6     CFKTNIDVIGDICLKGIEAHAESSEGQANVNFCLGVLAQKLGIGVKFCIVYANKQ-LSVK
Pr_Redulysin7     CKDCG-GFLGEVCIQSFEGRAKSSKGQANINFCLGILVKNLGFGIKGCFSYENKK-LSFK
Pr_Redulysin8     CCKLG-SFIGQLCLQGVEGRIKSSNGKPHVNLCAVALLKRFGVGAKICIGLEDGK-FKFK
Pr_Redulysin9     CCKLG-SFIGELCLQGVEGRLKSSNGKPHVNLCVGLLLKKFGCGAKICVSYVDGK-FSVS
Pr_Redulysin10    CLPPV-KVLGTPCVQAKAGVIKSSDGKSYLNMCAIAMLKNANACIKYCVTLENHK-IKFK
Pp_Redulysin1     CVKLG-SWFGKCCLQGVEGKITPK----SVNFCIAAVLKSYGVGAKLCLTHEDGK-TKVR
Pp_Redulysin2     CIKLG-SWFGKCCIQGVEGTIKAN----NINFCVAAVLKEYGVGAKICLNHENGK-LKVR
Pp_Redulysin3     CVNAG-SVIGKLCIKGVEGKGKSSQGQAQVNFCLGFLAEQHNVGAKVCATYENKK-FKIK
Pp_Redulysin4     CKNLG-KVLGDICLQGVEGRAKSSKGKPHINFCVAALLKTYGVGAKLCIGVEDGK-FKMR
Pp_Redulysin5     CLSLG-ELLGKVCVKCVEGRAKTSQGQGNVNFCLGLLAEKFSIGAKFCATYQDKK-LKIR
Pp_Redulysin6     CVNAG-SVIGKLCLKGMEGKGKSSQGKANVNFCLGFLAEKHNVGVKVCATYENKK-FKIK
Pp_Redulysin7     CLNAG-SVIGKICVKGVEGKGKSSQGKANVNFCLGFLAEKHNVGVKVCATYENKK-FKVK
Pp_Redulysin8     CLSLG-ELLGKVCVKCVEGRAKTSQGQGNVNFCLGLLAEKFSIGAKFCATYQDKK-LKIR
Pp_Redulysin9     CKNLG-KVLGDICLQGVEGRAKSSKGKPHINFCVAALLKTYGVGAKLCIGVEDGK-MKMR
Ti_Trialysin1     CVNVE-GFIGKVCMKGIEGHAKSSSGQANVNFCLGLVAEKFGVGAKLCGIYANKK-VRVK
Ti_Trialysin2     CVNVE-GFIGKVCMKGIEGHAKSSSGQANVNFCWGLVAEKFGVGAKLCGIYANKK-VRVK
Ti_Trialysin3     CVNVE-GFIGKVCMKGIEGHAKSSSGQANVNFCLGLVAEKFGVGAKLCGIYANKK-VRVK
Rp_Trialysin1     CVNLP-KPIKEVCIQDVW--VSVGQNGPNGDVCFTINFKSYSLGAKFCAGFNAASGFVPQ
Rp_Trialysin2     CVNLP-KPIKEVCIQNFW--VTVGQNGQNGDVCFTINFKSYSTGIKFCAGYTAQGGFAPH
                  *           *                   *              *
```

Supplementary Data 1. Amino acid sequence alignment of assassin bug redulysins together with homolog from kissing bug trialysins by MAFFT. Alignment of *R. iracundus* redulysin, together with African assassin bug *Platymeris rhadamanthus* (Pr_Redulysin), Australian assassin bug *Pristhesancus plagipennis* redulysin (Pp_Redulysin) and homolog toxin Trialysin from blood feeding *Triatoma infectans*, vector of *Trypanasoma cruzi*. Eight conserved cysteine residues are shown with asterisk (*).
