# Supplementary material for: Hexapod Assassins’ Potion: Venom Composition and Bioactivity from the Eurasian Assassin Bug Rhynocoris iracundus
Source: Biomedicines. 2021 Jul 14;9(7):819. doi: 10.3390/biomedicines9070819 (PMC8301361; doi:10.3390/biomedicines9070819)
Supplement: Supplementary file 1 [file biomedicines-09-00819-s001.zip › Supplementary Data S2 - Pair-wise identity matrix of Kininogen sequences.pdf]

	Ri-Kin	Pp-Kin1	Pp-Kin2	Pp-Kin3	Pp-Kin4	Pr-Kin1
**Ri-Kin**	**100.00**	58.41	50.45	51.35	32.14	38.94
**Pp-Kin1**	58.41	**100.00**	59.46	60.36	29.36	42.73
**Pp-Kin2**	50.45	59.46	**100.00**	80.18	31.13	38.98
**Pp-Kin3**	51.35	60.36	80.18	**100.00**	28.30	37.96
**Pp-Kin4**	32.14	29.36	31.13	28.30	**100.00**	37.27
**Pr-Kin1**	38.94	42.73	38.98	37.96	37.27	**100.00**

Supplementary Data 2: Pair-wise identity matrix of Kininogen sequences of assassin bug *R. iracundus* (Ri_Kin1), four Australian assassin bug *Pristhesancus plagipennis* (Pp_Kin) kininogens and one African assassin bug *Platymeris rhadamanthus* (Pr_Kin) kininogen.
