# Supplementary material for: Hexapod Assassins’ Potion: Venom Composition and Bioactivity from the Eurasian Assassin Bug Rhynocoris iracundus
Source: Biomedicines. 2021 Jul 14;9(7):819. doi: 10.3390/biomedicines9070819 (PMC8301361; doi:10.3390/biomedicines9070819)
Supplement: Supplementary file 1 [file biomedicines-09-00819-s001.zip › Supplementary Data S3 - Chitinase alignment.pdf]

```
R.iracundus-Hm  ------------------------------------------------MERNKNIKRLIY
P.plagipennis-Hm------------------------------------------------MERNKNIKRLLY
H.halys -Hm     ------------------------------------------------MEWRIGLSRLFC
C.lectularius-Hm------------------------------------------------MKRSISISRVFC
F.occidentalis-T----------------MHTLAQEATERSRKQPQPSQVP----------QMWSPGGPRALL
H.saltator-Hy   MGEQMLFPIDPTASLHLHFDLLTSALVSQREKVPTEVERKAIERLGLTIADEVSTGRTTW
A.cerana-Hy     ---------------------------------------------------MVSTSRTTC
                                                             .  *

R.iracundus     -LLLLCTLI-AIALSDSS-NTTRRRLRRP-----SGSSRVRDQETSASVNRFKV-RNR--
P.plagipennis   -LVLLCTLI-AIALTDSS-NTTRRRLRRP-----SGSSRVRDQETSASVNRFKV-RNR--
H.halys         -LLLLCVLL-TETYSESSTNTVRRRLRRP-----SGASRVRDQETSASVNRFKV-RNR--
C.lectularius   -LLLLCALI-SQTYTESS-TPTRRRLRRP-----SGSSRVRDQETSASVNRFKV-RNR--
F.occidentalis  -LAALALVL-LAQLTDSA-TGVRRRLRRPTNNHKTSSSPSADQEPSASVNRFKV-RSRLN
H.saltator      RLLLVCLVVLLLGLLQHT-EGARKRFRRPTT---SAPSISSDQEVSASVNRFKVTRNR--
A.cerana        WLLLVCLIV-LLGALRDA-EGTRKRFRRPAT---AAPSISNDQEVSASVNRFKVTRNR--
                 *  :. ::        :   .*:*:***     :..*   *** ********* *.*

R.iracundus     -----------VSQNSTPVISRKSSNDASSSSS--S--NEGLKVVCYYTNWSQYRLKVGK
P.plagipennis   -----------VSQNSTPVISRKSSNEASSSSS--T--NEGYKVVCYYTNWSQYRQKVGK
H.halys         -----------ISQNSTPIISRKSSNDGSSSSSKDS--KDGYKVVCYYTNWSQYRPKIGK
C.lectularius   -----------ISDKSTAVLTRKSGTDTASTSSSSSKNDDGYKVVCYYTNWSQYRPKIGK
F.occidentalis  AAAAGAAGAGAVATGAVVAASRKSSTVSNAVAPDSS----GYKVVCYYTNWSQYRPKVGK
H.saltator      -----------VSNKS-------SKNELQPGKT--S----DYKLVCYYTNWSQYRTKIGK
A.cerana        -----------ISNKS-------KKNEIQTDKS--E----DYKLVCYYTNWSQYRTKIGK
                           ::  :       . .   .  .       . *:*********** *:**

R.iracundus     FLPEDIDPDLCTHIIFAFGWLKKGKLSSFETNDETKDGKVGLYDRINNLKKANPKLKTLL
P.plagipennis   FLPEDIDPDLCTHLIFAFGWLKKGKLSSFETNDETKDGKVGLYDRINNLKKANPKLKTLL
H.halys         FLPEDIDPDLCTHIIFAFGWLKKGKLSSFESNDETKDGKIGLYEKIRNLKKANPKLKTLL
C.lectularius   FLPEDIDPNLCTHIIFAFGWLKKGKLSSFESNDETKDGKVGLYERIRNLKKANPKLKTLL
F.occidentalis  FTPEDIGADLCTHVIFAFGWLKKGKLSSFESNDETKDGKVGLYERIMTLKKANPKLKVLL
H.saltator      FLPEDIQPDLCTHVIFAFGWLKKNKLTSFESNDETKDGKTGLYERVVNLKKANPSVKVLL
A.cerana        FMPEDIQPDLCTHIVFAFGWLKKNKLTSFESNDETKDGKIGLYERVVNLKKANPSLKVLL
                * **** .:****::********.**:***:******** ***::: .******.:*.**
                                                      206
R.iracundus     AIGGWSFGTQKFKDMSATRYSRQTFIYSAIPFLRQRNFDGLDVDWEYPKGGDDKKNFVLL
P.plagipennis   AIGGWSFGTQKFKDMSATRYSRQTFIYSAIPFLRQRHFDGLDVDWEYPKGGDDKKNFVLL
H.halys         AIGGWSFGTQKFKDMSATRYSRQTFIYSAIPFLRGRSFDGLDVDWEYPKGGDDKKNFVLL
C.lectularius   AIGGWSFGTQKFKDMSSSRYARQTFIYSAVPFLRTRHFDGLDMDWEYPKGGEDKKNFVLL
F.occidentalis  AIGGWSFGTQKFKDMSKSRYARQTFIYSAIPFLRARNFDGLDMDWEYPKGTDDKKNFVLL
H.saltator      AIGGWSFGTQKFKDVSSTRYARQTFIYSAIPYLRDRNFDGLDIDWEYPKGGDDKKNYVLL
A.cerana        AIGGWSFGTQKFKDVSATRYARQTFIYSAISYLRDRNFDGLDIDWEYPKGGDDKKNYVLL
                **************:* :**:********:.:** * *****:******* :****:***

R.iracundus     LKELREAFEAEAQEVKKPRLLLSAAVPVGPDNVRSGYDVPAVAGYLDFINLMAYDFHGKW
P.plagipennis   LKELREAFEAEAQEVKKQRLLLSAAVPVGPDNVRSGYDVPAVAGYLDFINLMAYDFHGKW
H.halys         LKELREAFEAEAQEVKKPRLLLSAAVPVGPDNVRGGYDVPAVAGYLDFINLMAYDFHGKW
C.lectularius   LKELREAFEAEAQEVKKPRLLLSAAVPVGPDNVRGGYDVPAVAGYLDFINLMAYDFHGKW
F.occidentalis  LKELREAFEAEAQEVKKARLLLTAAVPVGPDNVKGGYDVPAVASYLDFINLMAYDFHGKW
H.saltator      LKELREAFEAEAQERKKPRLLLTAAVPVGPDNVKSGYDVPAVASYLDFINLMAYDFHGKW
A.cerana        LKELREAFEAEAQEKKMPRLLLTAAVPVGPDNVKSGYDVPAVASYLDFINLMAYDFHGKW
                ************** *  ****:**********:.********.****************

R.iracundus     ERETGHNAPLYAPSSDSEWRKQLSVSAAAEMWTRLGAPKEKLIIGMPTYGRTFTLTSIQK
P.plagipennis   ERETGHNAPLFAPSSDSEWRKQLSVSAAAEMWTRLGAPKEKLIIGMPTYGRTFTLTSLQK
```

```
H.halys           ERETGHNAPLFAPSSDSEWRKQLSVSAAAELWTRLGAPKEKLIIGMPTYGRTFTISTPKN
C.lectularius     ERETGHNAPLYAPSSDSEWRKQLSVSAAADMWTRLGAPKEKLIIGMPTYGRTFTLSSMQR
F.occidentalis    ERETGHNAPLYAPSSDSEWRKQLSVDYAANLWVRLGAPKDKLVIGMPTYGRSFTLSNTKQ
H.saltator        ERETGHNAPLYASSLDSEWQKQLSVDNAATMWVKLGAPKEKLIIGMPTYGRSFTLSNPAN
A.cerana          ERETGHNAPLYASSLDSEWQKQLSVDNAASMWVRLGAPKEKLIIGMPTYGRSFTLSNLSN
                  **********:*.* ****:*****. ** :*.:*****:**:********:**::.  .

R.iracundus       QTVNSPASGGGKAGEYTKEGGFLAYYEVCEMLRNGASYIWDDEMKVPYAIQGDQWVGFDD
P.plagipennis     LNVNSPASGGGKAGEYTKEGGFLAYYEVCEMLRSGAAYVWDDEMKVPYAIQGDQWIGFDD
H.halys           -TVNSPASGGGKAGEYTKEAGFLAYYEICEMLRNGAAYIWDDEMKVPYCIQGDQWIGFDD
C.lectularius     TSVNSPASGGGKAGEYTKEAGYLAYYEVCELLRNGASYIWDEEMKVPYAIQGDQWIGFDD
F.occidentalis    YKVNSPASGGGKAGEFTKEAGFLAYYEVCEMLKTGASYVWDDEMKVPYAVQGDQWVGFDD
H.saltator        FRVHAPANGGGKAGEYTKESGFLAYYEICEMLHNGAAYVWDDEMKVPYLVYNDQWVGFDD
A.cerana          FRVHAPASGGGKAGEYTKESGFLAYYEICEMLQNGAAYIWDDEMKVPYMVQNDQWVGFDD
                    *::**.*******:***.*:*****:**:*:.**:*:**:****** : .***:****

R.iracundus       EKSIRYKMKWLKESGFGGAMVWTVDMDDFNGTVCGNGVKYPLIGAIREELRGIKRGPNAQ
P.plagipennis     EKSIRYKMKWLKENSFGGAMVWTIDMDDFNGTVCGNNVKYPLIGAIREELRGIKRGPNAQ
H.halys           EKSIRYKMKWLKENGYGGAMVWTIDMDDFTGTVCGNDVKYPLIGAIREELRGIKRGPNAQ
C.lectularius     EKSIRHKSNWIKENGYGGAMVWTVDMDDFTGTVCGNGVKYPLIGAIREELRGVKRGPNAV
F.occidentalis    ERSIRHKMGWIKDNGFAGAMVWTVDMDDFTGTVCGGGVKYPLIGAIREELRGVSRGPNAK
H.saltator        EKSTRNKMRWLKSKGYGGAMVWTVDMDDFNGTVCGGNVRYPLIGAMREELRGISRGSSAK
A.cerana          EKSIRLKMDWLKSKGYGGAMVWTVDMDDFNGTVCGGNVKYPLIGAMREELLGISRGPNAK
                  *:* * *  *:*...:.******:*****.*****..*:******:**** *:.**..*

R.iracundus       DVDWTKVASSSSVAQVTKPVAIKIPVSDVLSRLNK--VKPTVSNAIIPIQDLNKREAQVF
P.plagipennis     DVDWTKVANSISV-EIAKPVAIKIPVSDVLSKLNK--VKPTVSNAIIPIQDLNKREAQVF
H.halys           DVDWEKVASSSKIVQPVKPQAIKIPVTELLSKLNK--AKPTVSNAILPIQDANTREAQVF
C.lectularius     DVDWTKVAKAPPPTA-VKPAAIKIPASDAISKLTR--AKPTVSNNIIPTQDANVREAQVF
F.occidentalis    DVDWSKVANTIVEEK-PLPSAVAIPVSEVIAKVRKPTKQATATATLTEVVDPNVRAPQVF
H.saltator        DIDWSAVATTITETIVKKPEPYKVSVSEVLSKSKK--LQK-STQLVINT-PIQEREAQTI
A.cerana          DVDWSAVATTVSETILKKPEPYKILVSDVINKAKK--HQKQTTQLVINL-PTNERPAQSM
                  *:**  **.:        *  .  : .:: : :  :   :   :  :      : * .* :

R.iracundus       CYLTSWSAKRPGGGRFSPSDLQPTLCTHVIYAFATLSDHKLAAAPGTE-EQYHKIVSLRE
P.plagipennis     CYLTSWSAKRPGGGRFSPSDLQPTLCTHVIYAFATLSDHKLAAAPGTE-EQYHKIVSLRE
H.halys           CYLTSWSAKRPGAGRFAPSDLQSNLCTHVIYAFATLTDHKLAAATGTE-DQYHKVVSLRD
C.lectularius     CYLTSWSAKRPGAGRFQPNDLQPNLCTHIIYAFATLTDHKLAAASGTE-EQYHKIIALRE
F.occidentalis    CYMTSWSQKRPGSGKFTPENIDASLCTHVIYAFATLKEHKLAMAEEKDADMYERVVALRE
H.saltator        CYLTNWSHKRPGMGRFNPEDIDPTLCTHVVYAFATLKEHLLTEGSDKDAEMYHRLIALRD
A.cerana          CYMTNWSHKRPGTGKFLPEDIDPTLCTHIVYSFATLKNYLLAEENEKDGEMYERLMTLRN
                  **:*.** **** *:* *.:::..****::*:****.:: *:         *.::::**:
                                                                 629
R.iracundus       KNPNLKILLAIGGWAFGSTPFKELTSNVFRMNQFVYEAIEFLREFQFNGLDVDWEYPRGI
P.plagipennis     KNPNLKILLAIGGWAFGSTPFKELTNNVFRMNQFVYEAIEFLREFQFNGLDVDWEYPRGI
H.halys           NNPNLKILLAIGGWAFGSTPFKELTSNVYRMNQFVYEAIEFLREYKFNGLDVDWEYPRGV
C.lectularius     QNPNLKILLAIGGWAFGSTPFKELTNNVYRMNQFVYEAIEFLREFKFNGLDVDWEYPRGV
F.occidentalis    KNPNLQILLAIGGWAFGSTPFKELTSNVFRMNQFVYEAIEFLREYKFNGLDVDWEYPRGA
H.saltator        KNPDIKILLAIGGWAFGSTPFKELTSNTFRMNQFVYEAIDFLREYKFDGLDVDWEYPRGA
A.cerana          KNPDLKILLAIGGWAFGSTPFRELTSNTFRMNQFVYEAIEFLREYKFDGLDVDWEYPRGG
                  :**:::***************:***.*.:**********:****::*:***********
```

```
R.iracundus      DDRASYVSLLRELRTAFEGEAKSSGQPRLLLTAAVPASFEAIAAGYDVPEIAKYLDFINV
P.plagipennis    DDRASYVSLLRELRTAFEGEAKTSGQPRLLLTAAVPASFEAIAAGYDVPEIAKYLDFINV
H.halys          DDRASYVSLLRELRTAFEGEAKTSGQPRLLLTAAVPASFEAIAAGYDVPEIAKYLDFINV
C.lectularius    DDRASYVNLLRELRMAFEGEAKSSGQPRLLLTAAVPASFEAIAAGYDVPEIAKYLDFINV
F.occidentalis   DDRAAYVNLLRELRMAFEGEAKSANLPRLLLSAAVPASFEAIAAGYDVPEISKYLDFINV
H.saltator       DDRSAYVNLLKELRLAFEGEAKSSGQPKLILSAAVPASFEAIAAGYDVPEISKYLDFINV
A.cerana         DDRAAYVNLLKELRLAFEGEANSSGQPKLILSAAVPASFEAIAAGYDVPEISKYLDFINV
                 ***::**.**:*** ******:::. *:*:*:*******************:********

R.iracundus      MTYDFHGQWERQVGHNSPLYPLESATSYQKKLTVDYSAREWVKQGAPKEKLMIGMPTYGR
P.plagipennis    MTYDFHGQWERQVGHNSPLYPLESATSYQKKLTVDYSAREWIKQGAPKEKLMIGMPTYGR
H.halys          MTYDFHGQWERQVGHNSPLYPLESATSYQKKLTVDYSAREWVKQGAPKEKLMIGMPTYGR
C.lectularius    MTYDFHGQWERQVGHNSPLYPLESATSYQKKLTVDYSAREWVKQGAPKEKLMIGMPTYGR
F.occidentalis   MTYDFHGQWERQVGHNSPLFPLESATSYQKKLTVDFSAREWVKQGAPKEKLMIGMPTYGR
H.saltator       MAYDFHGQWERQVGHNSPLYPLESATSYQKKLTVDYSAREWVKQGAPKEKLMIGMPTYGR
A.cerana         MTYDFHGQWERQVGHNSPLFPLESATSYQKKLTVDYSAREWVKQGAPKEKLMIGMPTYGR
                 *:*****************:***************:*****:******************

R.iracundus      SFTLVDETKFDIGAPASGGGKPGNYTAESGFMAYYEICDFLHEDNTTLVWDNEQQVPFAY
P.plagipennis    SFTLIDEAKFDIGAPASGGGKPGNYTAESGFMAYYEICDFLHEDNTTLVWDNEQQVPFAY
H.halys          SFTLVDEEKFDIGAPASGGGKSGNYTAESGFMAYYEVCDFLHEDNTTLVWDNEQQVPFAY
C.lectularius    SFTLVDEDKFDIGAPASGGGKPGNYTAESGFMAYYEVCDFLHEDNTTLVWDNEQQVPFAY
F.occidentalis   SFTLVDPTKFDIGAPASGGGTPGKYTGEAGFMSYYEVCDFLHGDNTTLVWDNEQQVPFAY
H.saltator       SFTLVDKEKFDIGAPASGGGVAGNFTNESGFLSYYEVCSFLNEDNTTLVWDSEQQVPFAY
A.cerana         SFTLVDKEKFDIGAPASGGGTSGNFTNESGFLSYYEICSFLNEDNTTLVWDSEQQVPFAY
                 ****:*  ************ .*::* *:**::***:*.**: ********.********

R.iracundus      RGDQWVGFDDERSLKTKMGWLKEEGFGGIMIWSIDMDDFRGVCGNGKFPLVNAMKQELED
P.plagipennis    RGDQWVGFDDERSLKTKMGWLKEEGFGGIMIWSIDMDDFRGVCGNGKFPLVNAMKQELED
H.halys          RGNQWVGFDDERSLKTKMGWLKEEGFGGIMIWSIDMDDFRGVCGNGKFPLVNAMKAELED
C.lectularius    RGNQWVGFDDERSLKTKMTWLKEEGFGGIMIWSIDMDDFRGICNNGKFPLVNAMKQELED
F.occidentalis   RDDQWVGFDDERSLKTKMTWLKDEGFGGIMIWSVDMDDFRGSCGTGKYPLMTAMRQELDG
H.saltator       RDDQWVGFDDERSLINKMSWLKEEGFGGVMIWSVDMDDFRGSCGSGRYPLIKAMKKELQG
A.cerana         RDDQWVGFDDERSLINKMQWLKEEGFGGIMIWSVDMDDFKGTCGAGKYPLIKAMMKELRD
                 *.:*********** .** ***:*****:****:*****:* *. *::**:.**  ** .

R.iracundus      YTISLEYQGPYEGTSTGGAYTTKDPNAVTCEEEDGHISYHPDKADCTMYYMCEGERKHHM
P.plagipennis    YSIKLEYQGPYEGTGTGGAYTTKDPNAVTCEEEDGHISYHPDKADCTMYYMCEGERKHHM
H.halys          YTIKLEYQGPYEGPSSSGAYTTKDPNAVTCEEEDGHISYHPDKADCTMYYMCEGERKHHM
C.lectularius    YSVKLEYQGPYEGTGSSGAYTTKDPNAVTCEEEDGHISYHPDKADCTMYYMCEGERKHHM
F.occidentalis   YKVKLEYDGPFEGTSTSGAYTTKDPNVVTCEEEDGHISYHPDKADCTRYYMCEGERKHHM
H.saltator       YKVKLEYDGPYESTSRNGKYTTKDPNEVMCDEEDNHISYHADKNDCTMYYMCEGERKHHM
A.cerana         YKVKLEYDGPYESHLRNGRYTTKDPNEVSCDEEDNHISYHPDKSDCTMYYMCEGERKHHM
                 *.:.***:**:*.   .* ******* * *:***.*****.** *** ************

R.iracundus      PCPSNLVFNPNENVCDWPENVEGCTQHTQAPPASRR-
P.plagipennis    PCPSNLVFNPNENVCDWPENVEGCTQHTQAPPASRR-
H.halys          PCPSNLVFNPNENVCDWPENVEGCTQHTQAPPPSR--
C.lectularius    PCPSNLVFNPNENVCDWPENVESCSQHTQAPPAARR-
F.occidentalis   PCPANLVFNPNENVCDWPENVEGCMHHTQAPPTSRRR
H.saltator       PCPVNLVFNPNENVCDWPENVEGCLQHTQAPPV----
A.cerana         PCPVNLVFNPNENVCDWPENVEGCLQHTQAPPV----
                 *** ******************.* :******
```

Supplementary Data 3: Alignment of Chitinases from order Hemiptera (Hm) (e.g. assassin bugs, bed bugs, stink bugs), Thysanoptera (T) (e.g. thrips) and order Hymenoptera (Hy) (e.g. ant and bee). Active regions were found using Interproscan website using *R. iracundus* protein sequence and highlighted in yellow. Asterisk (*) shows conserved amino acid residues. In bold are generally conserved residues from the active site motif known for the activity of glycosyl hydrolase family 18 (GH18).
