# Supplementary material for: Hexapod Assassins’ Potion: Venom Composition and Bioactivity from the Eurasian Assassin Bug Rhynocoris iracundus
Source: Biomedicines. 2021 Jul 14;9(7):819. doi: 10.3390/biomedicines9070819 (PMC8301361; doi:10.3390/biomedicines9070819)
Supplement: Supplementary file 1 [file biomedicines-09-00819-s001.zip › Supplementary Data S4 - Pair-wise identity matrix of Ptu1 sequences.pdf]

	Ri_Ptu1_1	Ri_Ptu1_2	Ri_Ptu1_3	Ri_Ptu1_4	Ri_Ptu1_5	Ri_Ptu1_6	Pr_Ptu1_1	Pr_Ptu1_2	Pp_Ptu1_1	Pp_Ptu1_2	Pp_Ptu1_3	Pt_Ptu1_1	MVIIA
Ri_Ptu1_1	**100.00**	35.85	26.53	15.91	38.46	30.00	47.92	50.00	83.64	26.92	33.96	35.48	27.45
Ri_Ptu1_2	35.85	**100.00**	31.91	20.45	37.25	33.33	43.75	42.31	35.85	28.85	78.18	35.48	24.49
Ri_Ptu1_3	26.53	31.91	**100.00**	30.23	44.00	30.77	30.95	30.61	26.53	79.63	34.04	45.45	45.45
Ri_Ptu1_4	15.91	20.45	30.23	**100.00**	33.33	28.26	23.08	25.00	20.45	25.58	22.73	32.26	31.11
Ri_Ptu1_5	38.46	37.25	44.00	33.33	**100.00**	31.37	36.96	37.25	38.46	38.00	41.18	32.26	36.17
Ri_Ptu1_6	30.00	33.33	30.77	28.26	31.37	**100.00**	25.58	36.73	26.00	32.69	31.25	36.36	27.78
Pr_Ptu1_1	47.92	43.75	30.95	23.08	36.96	25.58	**100.00**	42.55	45.83	27.66	45.83	31.03	25.00
Pr_Ptu1_2	50.00	42.31	30.61	25.00	37.25	36.73	42.55	**100.00**	53.70	27.45	48.08	35.48	22.00
Pp_Ptu1_1	83.64	35.85	26.53	20.45	38.46	26.00	45.83	53.70	**100.00**	28.85	33.96	32.26	27.45
Pp_Ptu1_2	26.92	28.85	79.63	25.58	38.00	32.69	27.66	27.45	28.85	**100.00**	30.77	42.42	22.45
Pp_Ptu1_3	33.96	78.18	34.04	22.73	41.18	31.25	45.83	48.08	33.96	30.77	**100.00**	38.71	20.41
Pt_Ptu1_1	35.48	35.48	45.45	32.26	32.26	36.36	31.03	35.48	32.26	42.42	38.71	**100.00**	30.00
MVIIA	27.45	24.49	45.45	31.11	36.17	27.78	25.00	22.00	27.45	22.45	20.41	30.00	**100.00**

Supplementary Data 4: Pair-wise identity matrix of Ptu1 family peptide sequences of assassin bug *R. iracundus* (Ri_Ptu1), Australian assassin bug *Pristhesancus plagipennis* (Pp_Ptu1), African assassin bug *Platymeris rhadamanthus* (Pr_Ptu1), *Peirates turpis* (Pt_Ptu1) and homolog toxin ‘’ω- conotoxins’’ (MVIIA) from a cone snail.
