# Supplementary material for: Hexapod Assassins’ Potion: Venom Composition and Bioactivity from the Eurasian Assassin Bug Rhynocoris iracundus
Source: Biomedicines. 2021 Jul 14;9(7):819. doi: 10.3390/biomedicines9070819 (PMC8301361; doi:10.3390/biomedicines9070819)
Supplement: Supplementary file 1 [file biomedicines-09-00819-s001.zip › Supplementary Data S5 - Accession numbers of the sequences.pdf]

Supplementary Data S5: Accession numbers of the sequences which are used for alignments and phylogenetic analyses.

Redulysins

Pr_Redulysin1: QHB21612.1
Pr_Redulysin2: QHB21549.1
Pr_Redulysin3: QHB21546.1
Pr_Redulysin4: QHB21541.1
Pr_Redulysin5: QHB21548.1
Pr_Redulysin6: QHB21542.1
Pr_Redulysin7: QHB21544.1
Pr_Redulysin8: QHB21545.1
Pr_Redulysin9: QHB21543.1
Pr_Redulysin10: QHB21547.1
Pp_Redulysin1: AQM58319.1
Pp_Redulysin2: AQM58384.1
Pp_Redulysin3: AQM58383.1
Pp_Redulysin4: AQM58381.1
Pp_Redulysin5: AQM58379.1
Pp_Redulysin6: AQM58385.1
Pp_Redulysin7: AQM58378.1
Pp_Redulysin8: AQM58386.1
Pp_Redulysin9: AQM58382.1
Ti_Trialysin1: AAL82380.1
Ti_Trialysin2: ABQ09358.1
Ti_Trialysin3: AAL82381.1
Rp_Trialysin1: BAV31387.1
Rp_Trialysin2: BAV31386.1

Kininogen

Pp_Kin1: AQM58342.1
Pp_Kin2: AQM58341.1
Pp_Kin3: AQM58343.1
Pp_Kin4: AQM58345.1
Pr_Kin1: QHB21503.1

Chitinase

Pp_Chi: ATU82720.1
Stingbug_Chi: XP_014294774.1
Cl_Chi: XP_014243752.1
Fo_Chi: XP_026279359.1
Hs_Chi: EFN89634.1

Ac_Chi: XP_016922880.1

Hemolysin

Pr_Hly1: QHB21514.1
Pr_Hly2: QHB21508.1
Pr_Hly3: QHB21510.1
Pr_Hly4: QHB21511.1
Pp_Hly1: ATU82699.1
Pp_Hly2: ATU82700.1
Pp_Hly3: ATU82704.1
Pp_Hly4: ATU82703.1
Pc_Hly: BBA30644.1
Ti_Hly: ABR27902.1
Ld_Hly: ATU82448.1

Ptu1 family peptides / Conotoxins

Pr_Ptu1_1: MN208349.1
Pr_Ptu1_2: MN208346.1
Pp_Ptu1_1: AQM58374
Pp_Ptu1_2: AQM58373
Pp_Ptu1_3: AQM58375.1
Pt_Ptu1_1: P58606
MVIIA: P05484.2
MVIIB: P05485.1
GVIIA/GVIIB: P05483.1
GVIA: P01522.2
CVID: P58920.1
