# Supplementary material for: Hexapod Assassins’ Potion: Venom Composition and Bioactivity from the Eurasian Assassin Bug Rhynocoris iracundus
Source: Biomedicines. 2021 Jul 14;9(7):819. doi: 10.3390/biomedicines9070819 (PMC8301361; doi:10.3390/biomedicines9070819)
Supplement: Supplementary file 1 [file biomedicines-09-00819-s001.zip › Supplementary Figure S1 - R. iracundus venom against Listeria grayi and Pseudomonas aeruginosa growth.pdf]

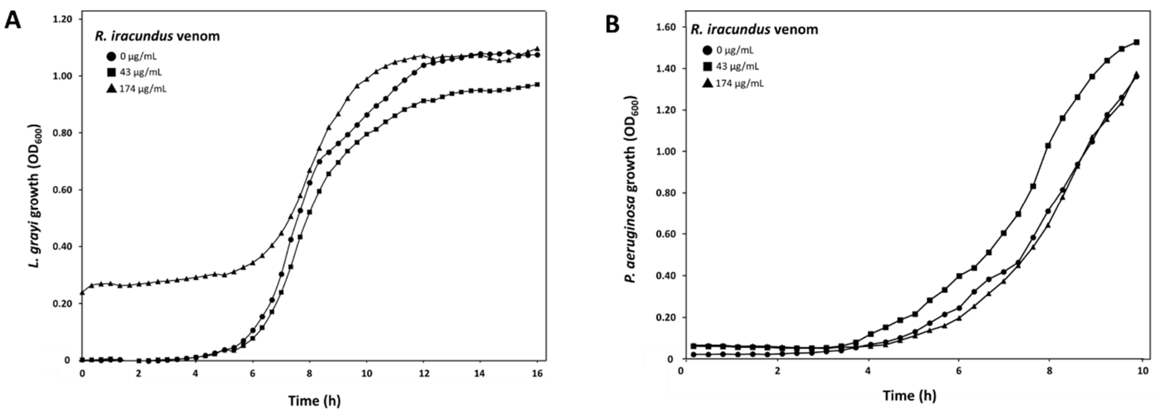

Figure S1. *Rhynocoris iracundus* venom against *Listeria grayi* and *Pseudomonas aeruginosa* growth. Venom activity was tested using different concentrations of crude venom incubated together with *Listeria grayi and Pseudomonas aeruginosa*. Bacterial growth of *L. grayi* and *P. aeruginosa* was monitored by measuring OD_{600} values at 20 min intervals for 16 h and 10 h, respectively. Venom did not inhibited the growth of both tested bacteria at tested concentrations (43 µg/mL and 174 µg/mL).
